# Integrated Analysis of Small RNA, Transcriptome and Degradome Sequencing Provides New Insights into Floral Development and Abscission in Yellow Lupine (*Lupinus luteus L.)*

**DOI:** 10.3390/ijms20205122

**Published:** 2019-10-16

**Authors:** Paulina Glazińska, Milena Kulasek, Wojciech Glinkowski, Waldemar Wojciechowski, Jan Kosiński

**Affiliations:** 1 Department of Plant Physiology and Biotechnology, Faculty of Biological and Veterinary Sciences, Nicolaus Copernicus University in Torun, 87-100 Torun, Poland; 2 Centre for Modern Interdisciplinary Technologies, Nicolaus Copernicus University in Torun, 87-100 Torun, Poland; 3 Department of Computational Biology, Institute of Molecular Biology and Biotechnology, Faculty of Biology, Adam Mickiewicz University, 61-712 Poznan, Poland

**Keywords:** yellow lupine, miRNA, phased siRNA, RNA-seq, degradome, flower development, abscission

## Abstract

The floral development in an important legume crop yellow lupine (*Lupinus luteus* L., Taper cv.) is often affected by the abscission of flowers leading to significant economic losses. Small non-coding RNAs (sncRNAs), which have a proven effect on almost all developmental processes in other plants, might be of key players in a complex net of molecular interactions regulating flower development and abscission. This study represents the first comprehensive sncRNA identification and analysis of small RNA, transcriptome and degradome sequencing data in lupine flowers to elucidate their role in the regulation of lupine generative development. As shedding in lupine primarily concerns flowers formed at the upper part of the inflorescence, we analyzed samples from extreme parts of raceme separately and conducted an additional analysis of pedicels from abscising and non-abscising flowers where abscission zone forms. A total of 394 known and 28 novel miRNAs and 316 phased siRNAs were identified. In flowers at different stages of development 59 miRNAs displayed differential expression (DE) and 46 DE miRNAs were found while comparing the upper and lower flowers. Identified tasiR-ARFs were DE in developing flowers and were strongly expressed in flower pedicels. The DEmiR-targeted genes were preferentially enriched in the functional categories related to carbohydrate metabolism and plant hormone transduction pathways. This study not only contributes to the current understanding of how lupine flowers develop or undergo abscission but also holds potential for research aimed at crop improvement.

## 1. Introduction

Yellow lupine is a crop plant with remarkable economic potential. Because of the symbiotic bond with nitrogen-fixing *Rhizobium* bacteria it does not need fertilizers, and its protein-rich seeds may be an excellent source of protein for both human consumption and animal feed [1,2,3]. *Lupinus luteus* flowers are stacked in whorls along the common stem forming a raceme. Pods are formed at the lowest whorls, while the flowers above them fall off [4]. The estimated percentage of dropped flowers is 60% at the 1st (and lowest) whorl, 90% at the 2nd whorl, and ~100% at the whorls above them. Thus, the problem of flower abscission generates large economic losses in agriculture [1]. Precise control of flower emergence and development is crucial for plant’s reproductive cycle. This is especially true for crop plants, as it is directly tied to potential yield. Molecular basis for flower formation has been extensively studied for many years across different plant species, and described collectively by ABCDE model (reviewed in [5]), with slight modifications depending on either species or flower shape [6]. Mutations that occur in genes governing flower formation cause various morphogenetic aberrations, including changes in the identity, number, and positioning of floral organs [7]. Proper development of already established flower elements is equally important. Numerous factors are involved in flower development, such as plant hormones (for example GA, IAA, JA [8]), numerous genes [9] and microRNAs, [10]. All of these components create a complex regulatory network, malfunction of which can cause a variety of abnormalities with the loss of fertility being the most detrimental [11,12].

Plant organ abscission is an element of the developmental strategy related to reproduction, defense mechanisms or disposal of unused organs [13,14]. In most species, the key components involved in the activation of the abscission zone (AZ) are plant hormones, in particular, auxin (IAA) and ethylene (ET) [15,16].

Our previous transcriptome-wide study [17] proved that the abscission of yellow lupine flowers and pods is associated, *inter alia*, with intensive changing of auxin catabolism and signaling. Genes encoding auxin response factors *ARF4* and *ARF2* were objectively more expressed in generative organs that were maintained on the plant, in contrast to the mRNA encoding auxin receptor *TIR1* (*TRANSPORT INHIBITOR RESPONSE 1*), which is accumulated in larger quantities in shed organs [17]. Since (i) some micro RNAs (miRNAs) and small interfering RNAs (siRNAs) restrict the activity of certain *ARFs* [18,19] and members of the *TAAR (TIR1/AFB AUXIN RECEPTOR)* family encoding auxin receptors [20], and since (ii) we proved that the precursor of miR169 is accumulated in increased quantities in yellow lupine’s generative organs undergoing abscission [17], we predict that sRNAs play significant roles in orchestrating organ abscission in *L. luteus*.

MiRNAs are 21-22-nt-long regulatory RNAs formed as a result of the activity of *MIR* genes in certain tissues and at certain developmental stages [21,22,23] and also in response to environmental stimuli [24,25,26]. *MIR* genes encode two consecutively formed precursor RNAs, first pri-miRNAs and then pre-miRNAs, which are subsequently processed by DCL1 (Dicer-like) into mature miRNAs [27,28]. *MIR* genes are often divided into small families encoding nearly or completely identical mature miRNAs [29]. miRNA sequences of 19–21 nucleotides are long enough to enable binding particular mRNAs by complementary base pairing, and allow either for cutting within a recognized sequence or for translational repression [30]. Plant miRNAs are involved in, for instance, regulating leaf morphogenesis, the establishment of flower identity, and stress response [10,24,25,26,31,32]. Some of them also form a negative feedback loop by influencing their own biogenesis, as well as the biogenesis of some 21-nt-long siRNAs called trans-acting siRNAs (ta-siRNAs). Ta-siRNAs are processed from non-coding *TAS* mRNAs, which contain a sequence complementary to specific miRNAs [33,34]. There is also a large group of plant sRNAs that are referred to as phased siRNA, which are formed from long, perfectly double-stranded transcripts of various origins, mainly processed by DCL4 [35,36].

Studies on sRNA in legumes (e.g., *Glycine max*, *G. soja* [37], *Medicago truncatula* [38], *M. sativa* [39], *Arachis hypogaea* [40], *Lotus japonicus* [41] and *Phaseolus vulgaris* [42]) have primarily focused on stress response or nodulation. Only three studies on miRNAs have been conducted so far using only two species of *Lupinus* genus: *Lupinus albus* (white lupine) and *Lupinus angustifolius* (narrow-leafed lupine). These studies were focused on small RNA sequences isolated from phloem exudate [43], global expression of miRNAs during phosphate deficiency [44], and gene regulatory networks during seed development [45]. Unfortunately, the knowledge on the roles of mi- and siRNAs function during flower development in leguminous plants is still incomplete [43]. Moreover, the involvement of regulatory sRNAs in mechanisms responsible for the maintenance/abscission of generative organs in the *Fabaceae* family has never been explored before.

Our observations of *L. luteus* generative development suggest that the fate of flowers (pod set or shedding) is determined on a molecular level during flower development. This study aims to characterize and investigate the role of these important molecules and their target genes during flower development and abscission.

In order to achieve this goal, an integrated analysis of small non-coding RNAs (sncRNA), transcriptome and degradome sequencing data was performed. We identified both known and presumably new miRNAs and siRNAs from flowers at different developmental stages, specifically the lower flowers (usually maintained and developed into pods) and the upper flowers (usually shed before fruit setting). Moreover, in our comparisons of libraries from the upper and lower flowers, differentially expressed miRNAs were found. In order to identify the miRNAs involved exclusively in flower abscission, we compared sRNA libraries from the pedicels of flowers that were maintained on the plant and those that were shed. A transcriptome- and a degradome-wide analysis was carried out to identify the target genes for the conserved or new *L. luteus* sRNAs. The targeted transcripts were then functionally annotated to outline the putative regulatory network in which these sRNAs might have a role to play. Our results of next-generation sequencing (NGS) analysis indicate that the identified miRNA-targeted modules may be vital in regulating yellow lupine flower development, both generally and depending on the flower location on the inflorescence. Furthermore, these scnRNA also display differential accumulation during flower abscission in this plant.

## 2. Results

### 2.1. Sequencing and Annotation of Yellow Lupine sRNAs from Flowers and Flower Pedicels

**Figure 1:**
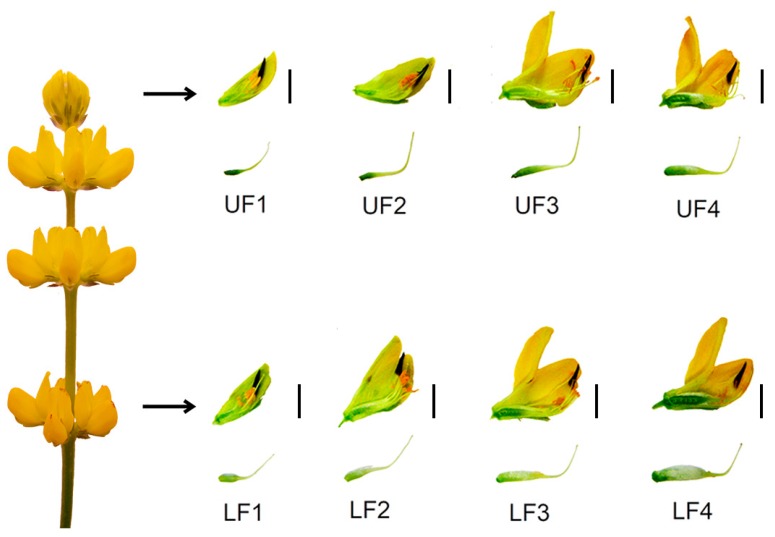
Development of *Lupinus luteus* flowers from the upper and lower part of the raceme. An isolated pistil from a given developmental stage is shown under each flower. LF—lower flower, UF—upper flower. Bar 5 mm.

**Figure 2:**
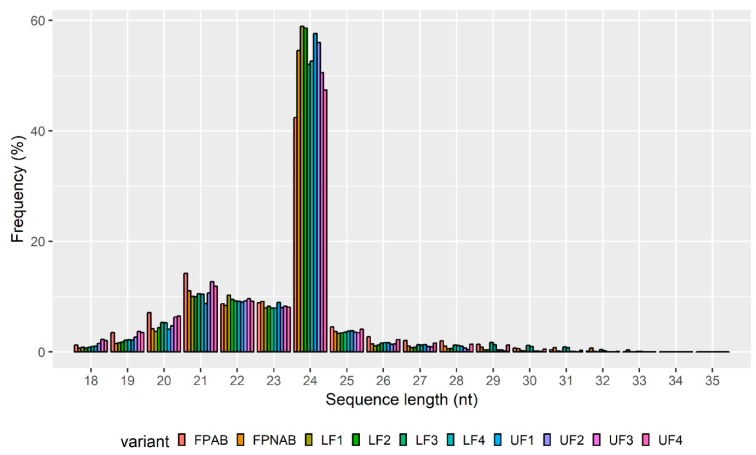
Nucleotide length distribution of small RNAs from all ten libraries: *Y*-axis represents the percentage frequency of the sRNA sequences identified in this study, the *X*-axis represents sRNA length.

Flowers collected from the top and bottom parts of the inflorescence were separated into four categories based on the progression of their development, and thus: Stage 1—closed green buds, parts of which were still elongating. Stage 2—closed yellow buds, around the time of anther opening. Stage 3—flowers in full anthesis. Stage 4—flowers with enlarged gynoecia from the lower parts of the inflorescence, or aging flowers from the upper parts of the inflorescence. Based on their position on the inflorescence, flowers in each of the stages were additionally tagged as either upper (UF) or lower flowers (LF), resulting in eight different variants: UF1, UF2, UF3, UF4, LF1, LF2, LF3 and LF4 (Figure 1, Table S1). Flower pedicels from flowers undergoing abscission (FPAB) or maintained on the plant (FPNAB) were also collected, as they had been in our previous study [17]. This division resulted in ten variants of small RNA libraries, which were subjected to single-end deep sequencing performed on the Illumina HiSeq4000 platform (Illumina, Great Abington Cambridge, United Kingdom). After removing low-quality reads, a total of 303,267,263 reads (from 14,186,278 to 15,504,860 reads per library) and 128,060,403 unique reads (from 5,677,701 to 6,990,061 per library) were obtained (Table S2).The length distribution of the small RNAs (15–30 nt) revealed that a length of 24 nt was the most frequent and that of 21 nt was the second most abundant class of the clean and redundant reads (Figure 2), which was compliant with many other RNA-Seq experiments [46,47,48] and correlated with the abundance of siRNAs and miRNAs, respectively.

The unique reads were annotated against Rfam [49,50] and miRBase [51] databases, and from the latter both mature (named in tables as ‘miRBase’) and precursor sequences (named as ‘Hairpin’) were taken into account. However, many of them remained unassigned (Table 1).

The unique sequences were annotated into different RNA classes against the Rfam database using BLAST [52] such as known miRNAs, rRNA, tRNA, sn/snoRNA and others (Table 2). A total of 690,436 sRNAs were annotated into all libraries, with the highest number observed in the upper flowers and abscising pedicles. Between these libraries, the most abundant classes were rRNAs and tRNAs, with average values of 26,390 and 3,726 sequences, respectively, followed by snoRNAs and different subtypes of snRNAs with average values ranging from 863 to 876 sequences (Table 2).

### 2.2. Identification of Known, Conserved miRNAs

**Figure 3:**
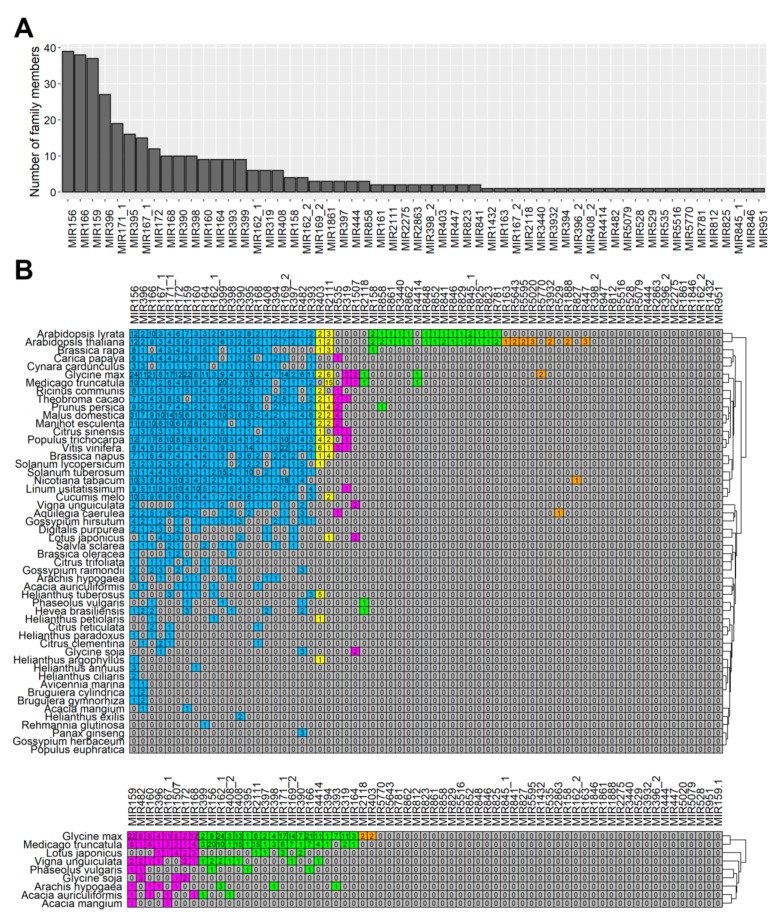
Identification and evolutionary conservation of known miRNA families in *Lupinus luteus*. (**A**) The distribution of known miRNA family sizes in *L*. *luteus*. (**B**) Comparison of known miRNA families in *L*. *luteus* and their 52 homologs in *Eudicotyledons* species present in miRBase (upper panel) and 9 *Fabaceae* species (lower panel). Known miRNA families of *L*. *luteus* identified from small RNA-seq are listed in the top row. The colors represent relative miRNA families classified into different groups with similar conservation. Blue, yellow, magenta, green and orange represent relative miRNA families with homologs across more than 20, 10–19, 5–9, 2–4 species and in 1 species, respectively.

After analyzing the results of the alignment against miRBase [51], 394 unique miRNAs containing 366 conserved miRNAs were identified (Table S2). The number of identified miRNAs in each library is shown in Table S1. The identified miRNAs belonged to more than 67 families (Table S2), while most of them belonged to the MIR156, MIR159, and MIR166 families, with more than 35 members in each (Figure 3a). Each discovered miRNA received an identification number in the following format: Ll-miR(number). In case of miRNAs displaying identity to sequences from miRBase, annotation Ll-miR(number)/miRBase annotation is used, for example, Ll-miR224/miR393.

### 2.3. Evolutionary Conservation of microRNAs Identified in Lupinus luteus

Since this study is the first wide-scale analysis of yellow lupine miRNAs, we decided to explore the evolutionary characteristics of these sequences when compared to the data of almost all [52] *Eudicotyledons* species present in miRBase [51]. The same analysis was performed exclusively against nine *Fabaceae* species. As shown in Figure 3b, the 67 known miRNA families exhibited different numbers of homologous sequences in both of the comparisons. Twenty of them were the most conserved ones, i.e., had homologues in over 20 species (Figure 3b, shaded in blue). Our comparison across legumes revealed that 8 miRNA families were highly conserved in this taxon, i.e., had homologues in 5–9 species out of 9 (Figure 3b, lower panel, shaded in magenta), 18 had homologues in 2–4 legumes (Figure 3b, shaded in green), and 2 had homologues only in one plant, *Glycine max* (Figure 3b, shaded in orange).

A surprisingly high number (39) of miRNA families identified in yellow lupine flowers were not conserved across *Fabaceae*, probably due to a still incomplete list of miRNAs in these taxa.

### 2.4. Identification of Novel miRNAs

With the use of the ShortStack software (https://github.com/MikeAxtell/ShortStack/) [53], 28 candidates for novel miRNAs were identified (Table 3). This tool identifies miRNAs based on their mapping against a reference genome. Since there was no genome available for the studied species, we used a transcriptome instead (statistical data on de novo assembly is shown in Table S3). The results obtained were filtered against mature miRNAs from miRBase, and unique sequences received names in the following format: “Ll-miRn(number)”, (for example, Ll-miRn1). All of these 28 sequences were 21–24 nt in length, with 68% of them being 21 nt long (Table 3).

The expression of novel miRNAs was also highly diversified across all the libraries. Ll-miRn26 was present only in the LF1 sample, while Ll-miRn21 was present in all the sRNA libraries and had an expression ranging from 3,982.82 to 11,421.55 RPM (Table S4).

### 2.5. Analysis of the Expression Abundance of Known miRNA Families

**Figure 4:**
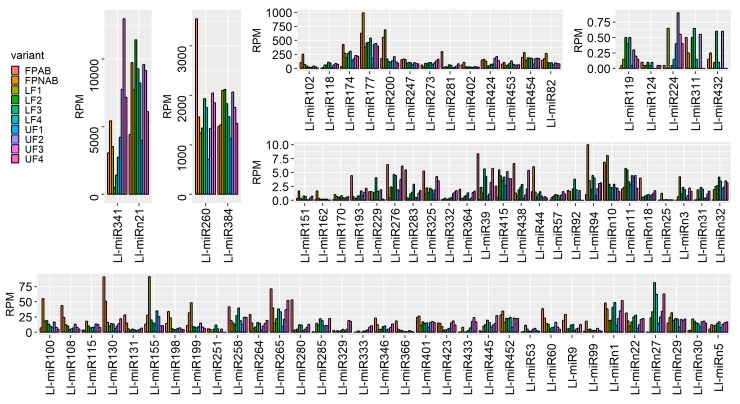
Diversity of miRNA expression (reads per million, RPM) in yellow lupine flowers. Complete data concerning differential miRNA expression in the experiment described herein, divided into six groups, depending on their expression maxima listed in order of appearance from left to right, and top to bottom: over 10,000 RPM, 2000–10,000 RPM, 100–2000 RPM, up to 1 RPM, 1–10 RPM, 10–100 RPM.2.6. Identification of phased siRNA in Yellow Lupine.

Since miRNA expression across all libraries displayed high variation, we put the data into five categories based on the maximum value (Figure 4). Two miRNAs, namely Ll-miR341/miR319 and Ll-miRn21, showed expression maxima of over 10,000 RPM. The maximum expression of another two, Ll-miR260/miR166 and Ll-miR384/miR396, ranged from 2000 to 10,000 RPM. Thirteen miRNAs showed expression maxima ranging from 100 to 2000 RPM. The most numerous category, with 33 elements was the one for miRNAs with expression maxima ranging from 10 to 100 RPM. Another 24 miRNAs were expressed with the maximal RPM values between 1 and 10. The expression value of the five least abundant miRNAs did not exceed 1 RPM (Figure 4, Table S5).

Numerous reports and studies indicate the importance of phased siRNA not only in stress response mechanisms but also in growth regulation [54]. Therefore, we decided to investigate the role of siRNAs during yellow lupine inflorescence development. To achieve this, ShortStack (https://github.com/MikeAxtell/ShortStack/) [53] was used to identify small RNAs that were being cut in phase from longer precursors. We identified 316 siRNA ranging from 21 to 25 nt in length, of which 71% were 24 nt long (Table S6, Figure S1). The identified siRNAs received names in the following format: “Ll-siR(number)”, (for example Ll-siR1) and displayed a highly differential expression pattern (Table S7). Some of the sequences showed organ-specific expression, for example, Ll-siR4, -13, -173 were present only in the pedicels of abscising flowers (FPAB), while Ll-siR308 showed an elevated expression in the pedicels (FPAB and FPNAB). On the other hand, Ll-siR246, -291 and -56 were present almost exclusively in the youngest flowers in the lower part of inflorescence (LF1) (Table S7).

### 2.6. Analysis of the Expression Profile of the Identified sRNAs During Yellow Lupine Flower Development

**Figure 5:**
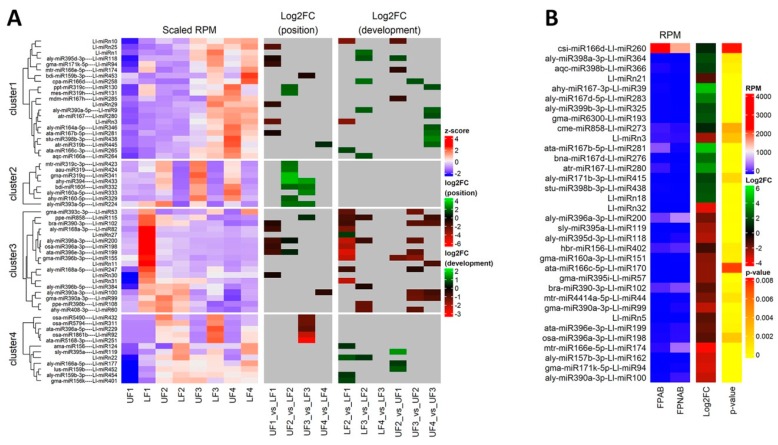
Differential miRNA expression in lupine flowers and flower pedicels. (**A**) Heatmaps of z-scaled miRNA expression (scaled RPM) and log_2_ fold changes for either position of the flower on the raceme (Log_2_FC position) or identified between consecutive stages of flower development (Log_2_FC development). Grey indicates insignificant changes. (**B**) Heatmaps of miRNA expression, log_2_ fold changes (Log_2_FC) and *p*-values for flower pedicels with an active or inactive abscission zone. The miRNA names are shown on the right vertical axis. Red and green represent the up-regulated and down-regulated miRNAs, respectively.

To gain better insight into the dynamics expression of the identified sRNAs during floral development in yellow lupine, we established a wide scope comparison of the following growth stages of flowers from the upper (UF2 vs UF1, UF3 vs UF2 and UF4 vs UF3) and lower (LF2 vs LF1, LF3 vs LF2 and LF4 vs LF3) parts of the inflorescence (Table 4, Figure 5).

The analyses resulted in the identification of 30 differentially expressed miRNAs (DEmiRs) in the lower and 29 in the upper flowers (Table 4), as well as 14 and 7 DE siRNAs, respectively (Table S8). Between UF2 and UF1, there was a change in the expression of 8 miRNAs, 2 sequences belonging to MIR359 and MIR166 families each, as well as one representative of each of the MIR159, MIR167, MIR396 families and novel Ll-miRn10. Ten DE miRNAs were identified in a comparison of UF3 vs UF2, of which only one Ll-miR258/miR166 was up-regulated. The remaining miRNAs were downregulated and consisted of 3 sequences belonging to the MIR390 and MIR396 families each, and single miRNAs from the MIR168, MIR408, and MIR396 families. A comparison of the UF4 vs UF3 libraries revealed 12 DEmiRs. The most numerous group were members of the MIR390 family, followed by 2 members of MIR167 and MIR319, and singular representatives of MIR398, MIR164, and MIR858, with one novel Ll-miRn11 (Table 4).

During the development of flowers from the lower part of the inflorescence, the miRNAs accumulation dynamics were different. The highest number of the identified DEmiRs was found comparing the youngest flowers (LF2 vs LF1), while, interestingly, a complete lack of DE miRNAs was found when comparing the oldest flowers: LF4 vs LF3 (Table 4). In our comparison of LF2 vs LF1, among the 18 DEmiRs, the most numerous group were novel miRNAs, followed by members of the MIR396 family. Between the LF2 and LF3 stages we confirmed that there was a change in the accumulation of 11 miRNAs, and this pertained to two members of the MIR166 and MIR399 families each, Ll-miRn1 and Ll-miRn22, which were followed by single representatives of the MIR390, MIR395, MIR858, MIR398, MIR408 families (Table 4).

**Figure 6:**
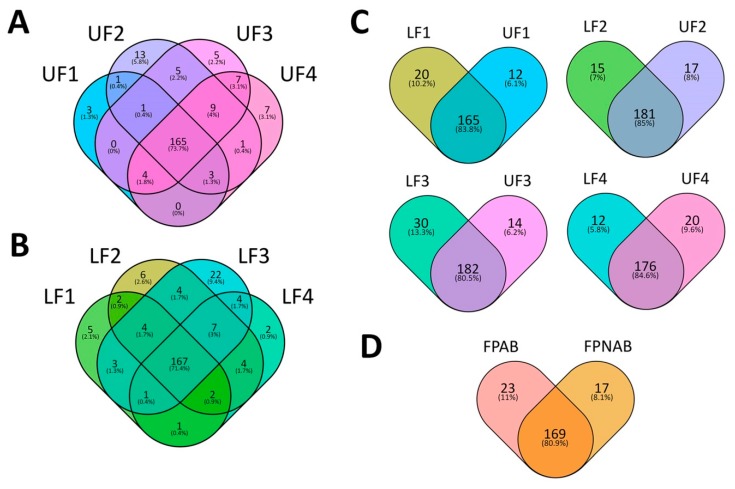
Diagrams showing distribution of yellow lupine miRNAs in (**A**) upper flowers, (**B**) lower flowers, (**C**) both upper and lower flowers at particular stages of their development, (**D**) pedicels of abscising flowers or flowers maintained on the plant.

In order to identify miRNAs the presence of which is either common or unique depending on the developmental stage of the upper and lower flowers in lupine, Venn diagrams were constructed (Figure 6a and Table S9) using Venny 2.1 (https://bioinfogp.cnb.csic.es/tools/venny/) [55]. The results of these analyses revealed that approximately 70% of the identified miRNAs were common in all developmental stages of both the upper (Figure 6a) and lower flowers (Figure 5b). However, miRNAs unique to certain developmental stages were also found (Figure 6 and Table S9).

In regard to siRNAs during flower development in yellow lupine, almost every differentially expressed siRNA was up-regulated. In the lower part of the inflorescence, similarly to miRNAs, there were no differences between the LF4 and LF3 stages. During the upper flower development, most DEsiRs were identified in a comparison of UF2 vs UF1, and the least (only one) when comparing UF3 vs UF2. One noteworthy observation was the presence of the same siRNAs in the comparisons of UF2 vs UF1 and LF2 vs LF1, namely Ll-siR281, -308. and -249, which suggests that an increase in their accumulation is important during phase 1 to phase 2 transition in the development of yellow lupine flowers, regardless of their position on the inflorescence. The complete dataset can be found in (Table S8).

### 2.7. Comparison of Differentially Expressed sRNAs Between Developing Flowers From the Lower and Upper Whorls of the Raceme

In order to determine the differences in sRNA expression in developing yellow lupine flowers, comparative analyses of both the upper and lower flowers were performed for each developmental stage of the inflorescence (LF1 vs UF1, LF2 vs UF2, LF3 vs UF3 and LF4 vs UF4) (Table 5, Figure 5). In general, 46 DEmiRs were identified (Table 5). In the first stage of development, the most numerous group of DEmiRs was that of the novel sequences (Ll-miRn3, -25, -29 and -30), followed by sequences annotated as miR396 (3 miRNAs). In the second stage of flower development, miRNAs belonging to the MIR319 family were identified as the largest group (5 sequences), followed by two DE miRNAs annotated as miR160 (Ll-miR329/miR160-5p, Ll-miR332/miR160f) and miR396 (Ll-miR199/miR396e-3p, Ll-miR200/miR396a-3p), respectively. The third stage turned out to be the most diverse, with 2 representatives of the MIR160 (Ll-miR333/miR160a-5p and Ll-miR332/bdi-miR160f) family, followed by single sequences annotated as Ll-miR433/miRr394, Ll-miR224/miR393a-5p, Ll-miR115/miR858, Ll-miR453/miR19b-3p, Ll-miR229/miR396a-5p, Ll-miR432/miR490 and Ll-miR92/miR5168-3p.

Regarding the phased siRNAs, only 4 of them displayed differential expression, namely Ll-siR119 at stage 1 and Ll-siR224, -100 and -146 at stage 4. These results might suggest that, firstly, miRNAs display differential expression in each and every stage of flower development, regardless of flower position on the inflorescence, and secondly, that miRNAs seem to be much more impactful in comparison with phased siRNA in regards to yellow lupine flower differentiation.

Analyses of the Venn diagrams we created (Figure 6c), displaying the presence profiles for the library miRNAs, revealed that in each comparison between the upper and lower flowers (UF1 vs LF1, etc.) around 80% of the identified sequences were common for both the upper and lower flowers (Figure 6c). However, in each comparison, we were able to identify miRNAs unique to each stage of the development and each flower position. For example, 20 miRNAs were exclusively present in LF1, while 12 miRNAs were unique to UF1. The detailed information on these comparisons can be found in Table S9.

Based on the data received, we suggest that differences in miRNA expression between lower and upper flowers may be related to the fate of these organs (pod formation/flower abscission). To further confirm this function, we performed an experiment in which flowers were removed from the lower whorls, leaving only flower buds from the last, top whorl (Figure S2). Removing the lower flowers causes maintenance of flowers on the plant and their development into pods, unlike flowers from this whorl in control plants. Thus, their fate seems to be associated with the location in the inflorescence changes. Then, the expression of selected lupine DEmiRs and their target genes were compared during the development of upper flowers after removal of the lower flowers (UFR) in the development stages of S1-S4, with control upper (UF S1–S4) and lower (LF S1–S4) flowers, respectively (Figure S3). The obtained results show that the removal of lower flowers caused a change in the levels of chosen sRNAs in upper flowers and it similar in this respect to flowers from the lower part of raceme. This indicates a link between these genes and the fate of the flowers.

### 2.8. Comparison of Differentially Expressed sRNAs Between Flower Pedicels with Active And Inactive Abscission Zones

To identify sRNAs possibly involved in yellow lupine flower abscission, mi- and siRNA expression patterns for flower pedicels with an active abscission zone (AZ) (FPAB) and inactive AZ (FPNAB) were compared. As a result, 34 DE miRNAs (including 5 novel ones) (Table 6) and 20 DE phased siRNAs (Table S8) were identified. 14 miRNAs and 9 siRNAs were up-regulated, while the rest remained down-regulated in FPNAB. Among the up-regulated miRNAs, the most numerous family was MIR167 (5 members), followed by MIR398 (3 members). Among the down-regulated miRNAs, the most abundant were MIR390, MIR396 and MIR395 families with 3 members each (Table 6, Figure 5b). With regard to siRNAs, the most up-regulated in FPANB were Ll-siR173, -4 and -13, and the most down-regulated was Ll-siR208 (Table S8).

An analysis of the Venn diagrams based on the presence of the identified miRNAs revealed that approx. 80% of the miRNAs were present in both abscising and non-abscising flower pedicles (Figure 6d). However, 23 miRNAs remained unique to FPAB and 17 to FPNAB (Figure 6d, Table S9).

### 2.9. Validation of the Identified sRNAs in RNA-seq

**Figure 7:**
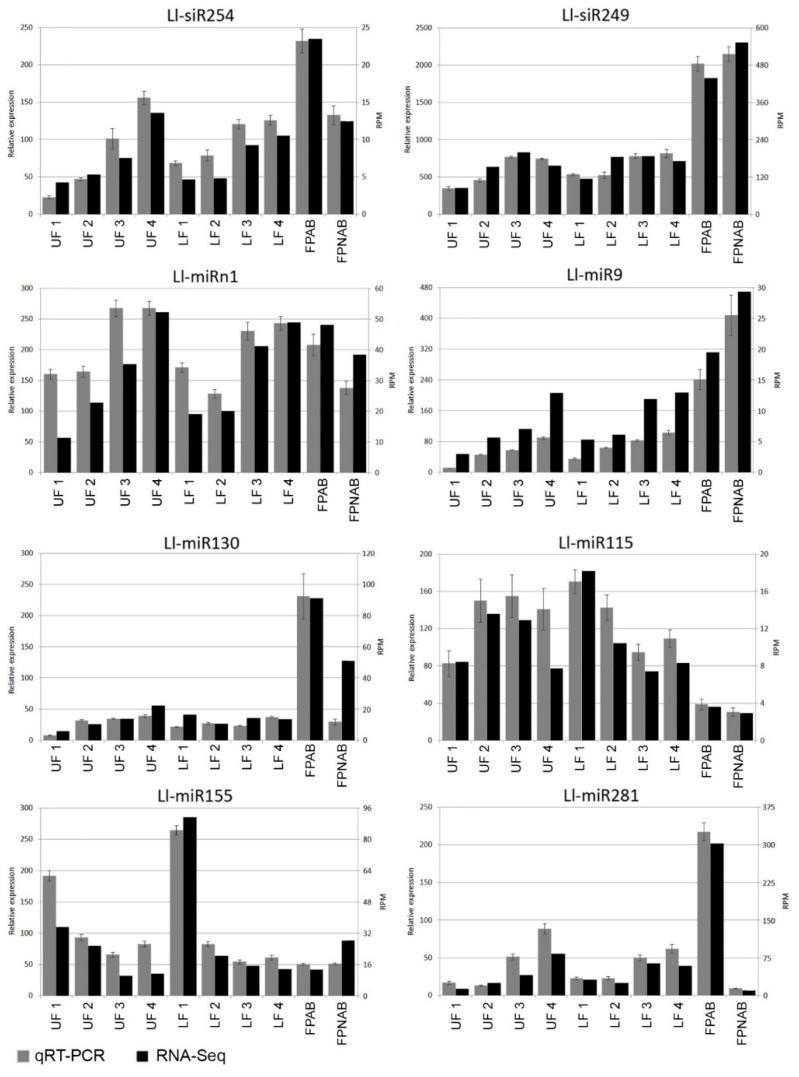
SL RT-qPCR validation of selected sRNAs in *L*. *lupinus*. Grey indicates the miRNA expression levels determined by qPCR. Black indicates the miRNA expression levels determined by deep sequencing. Vertical bars indicate standard errors.

Stem-loop RT-qPCR technique [56,57] was employed in order to validate the data generated using deep sequencing technology and to confirm the expression patterns of the identified sRNA. Eight identified sRNAs (six conserved miRNAs, one novel miRNA, and one siRNA) were used for this task (Table S10). The qPCR results were similar to sRNA-seq data (Figure 7). For example, in the RT-qPCR analysis, the Ll-siR254 expression increased as the flower developed, showing a positive correlation with the deep sequencing results. Ll-siR249 was preferentially accumulated in yellow lupine pedicels, both in qPCR and RNA-seq. The results of the expression analysis of these sRNAs supported the validity of our sRNA-Seq.

### 2.10. Identification of sRNA Target Genes using Degradome and psRNATarget Analysis

**Figure 8:**
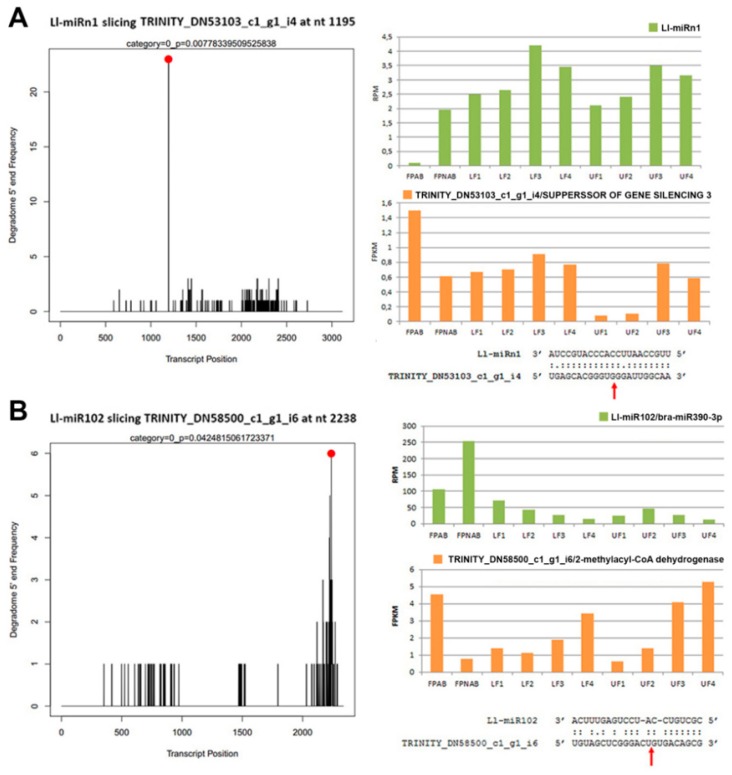

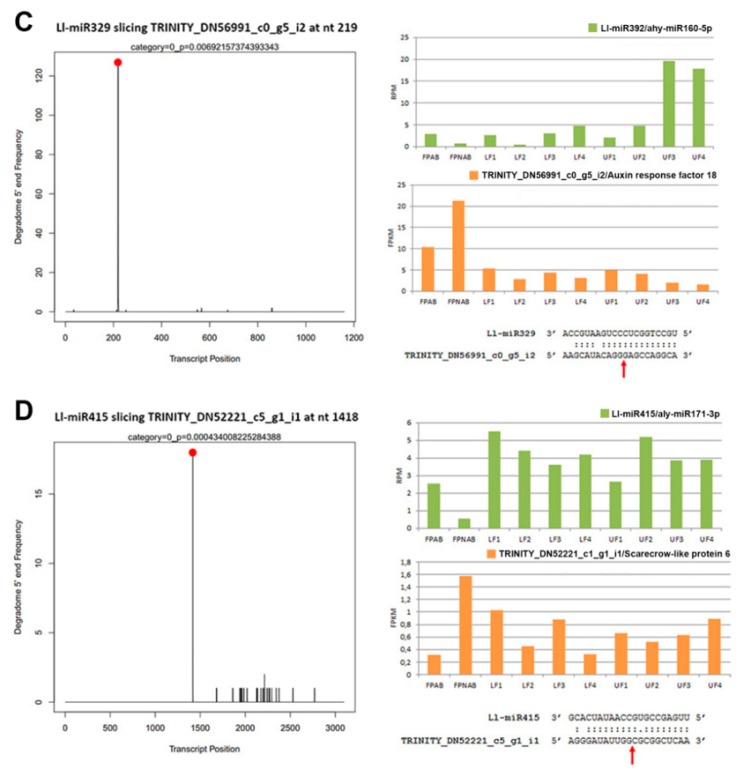
Examples of post-transcriptional regulation of miRNA targets in yellow lupine. (**A**) Ll-miRn1 and *SGS3* mRNA, (**B**) Ll-miR102 and *2-methylacyl-CoA dehydrogenese* mRNA, (**C**) Ll-miR392 and *ARF18* mRNA, (**D**) Ll-miR415 and *SCL6* mRNA. The T-plots show the distribution of the degradome tags along the full length of the target gene sequence. The cleavage site of each transcript is indicated by a red dot. Comparison of the expression levels of miRNAs and their targets in flowers from upper and lower whorls of yellow lupine racemes, and flower pedicels, as determined by deep sequencing. In miRNA-mRNA alignments, the red arrows indicate the cleavage site of the target gene transcript.

In order to estimate accurately the biological function and impact of certain miRNAs, their target genes needed to be identified. To achieve this, we constructed degradome libraries from pooled samples of stage 3 upper and lower parts of the inflorescence. Through total degradome library sequencing, 19,353,278 raw reads were obtained (Table S11). After quality filtering, the degradome data were aligned to the reference transcriptome with CleaveLand 4 [58] to find sliced miRNA and siRNA targets. After processing and analysis, a total of 14,077 targets were identified, and after filtering with a *p*-value < 0.05, 538 targets emerged (501 targets for 178 known miRNAs and 37 targets for 13 novel miRNAs) (Table S12). For the phased siRNAs, 3,340 targets were initially identified, and after similar filtering, their number dropped to 89 targets for 46 siRNAs (Table S13). Exemplary target t-plots and sequences of the miRNAs and target mRNAs are shown in Figure 8.

As expected, many of the targets for evolutionarily conserved miRNAs were compliant with literature data. For example, Ll-miR329/miR160-5p targeted *ARF16* and *ARF18*, the Ll-miR415/miR171b targeted *SCL6*, Ll-miR341/miR319q targeted *TCP2*, Ll-miR224/miR393a-5p targeted *TIR1*, etc. (Table S12).

A comparison of the expression of four exemplary miRNAs and their target genes (Figure 8) confirmed the reverse-correlation in the accumulation of miRNAs and an abundance of mRNA target genes, especially in flower pedicels. In the flowers, this correlation was not so obvious, presumably because of the organ’s more complex nature (with its various elements, such as the stamen and the pistil), where regulation could be tissue specific.

In the case of some of the identified mi- and siRNAs, we were unable to determine the targets with a degradome analysis, which might have been caused by the lack of a sufficient amount of cleavage products ensuing from using only stage 3 flowers to construct the library. In order to find the putative missing target genes, the psRNATarget tool [59] was employed, which rendered plausible target genes through a comparison of the sRNAs with the reference transcriptome containing data from all of the samples. Using this method, we managed to establish putative target genes for most of the mi- and siRNAs, obtaining 66,102 miRNA and 32,725 siRNA targeted transcripts. A full list of the targets identified using the psRNATarget or degradome analysis for DE miRNAs, siRNAs, and novel miRNAs is contained in Table 3, Table 4, Table 5, Table 6 and Table S13. Targets for all of them are shown in Tables S14, S15, S16, S17 and S18.

### 2.11. Function of the miRNAs Potential Targets

**Figure 9:**
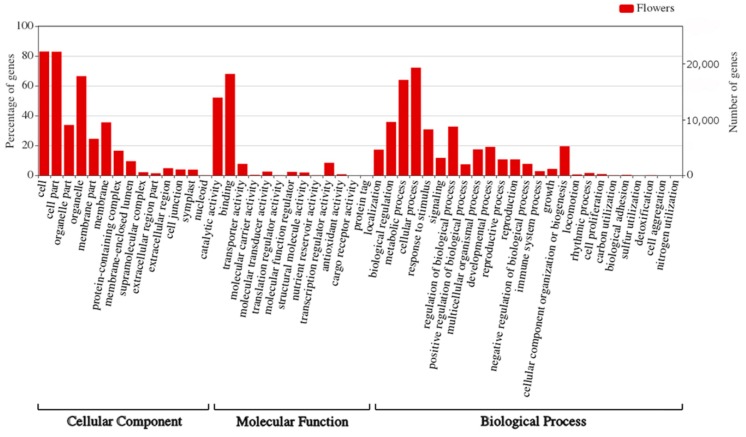
Visualization of GO categories annotated to predicted target genes of known and novel miRNAs in yellow lupine.

Gene Ontology (GO) analysis was performed in order to investigate the functions of the miRNAs targets identified in yellow lupine flowers. Among the 27,547 targets of known and novel miRNAs identified with psRNATarget 26,230 targets exhibited GO terms (Table S17). 23,092 genes were categorized into ‘Cellular component’, 23,501 into ‘Molecular function’, and 22,939 into ’Biological process’. Figure 9 shows target gene percentages for each GO category. The largest number of targets classified as ‘Cellular component’ was attributable to ‘cell’, ‘cell part’ and ‘organelle’. The majority of targets of the ‘Molecular function’ category were classified as ‘binding’ and ‘catalytic activity’. Within the ‘Biological process’, most of the targets were categorized as ‘cellular’ and ‘metabolic process’ (Figure 9, Table S17). Within the ‘Flower development’ category, the targets of 37 miRNAs fit within GO terms related to phytohormones (Figure S4a), and the targets of 69 miRNAs were placed into the category of GO terms related to the development of flower parts (Figure S4b).

**Figure 10:**
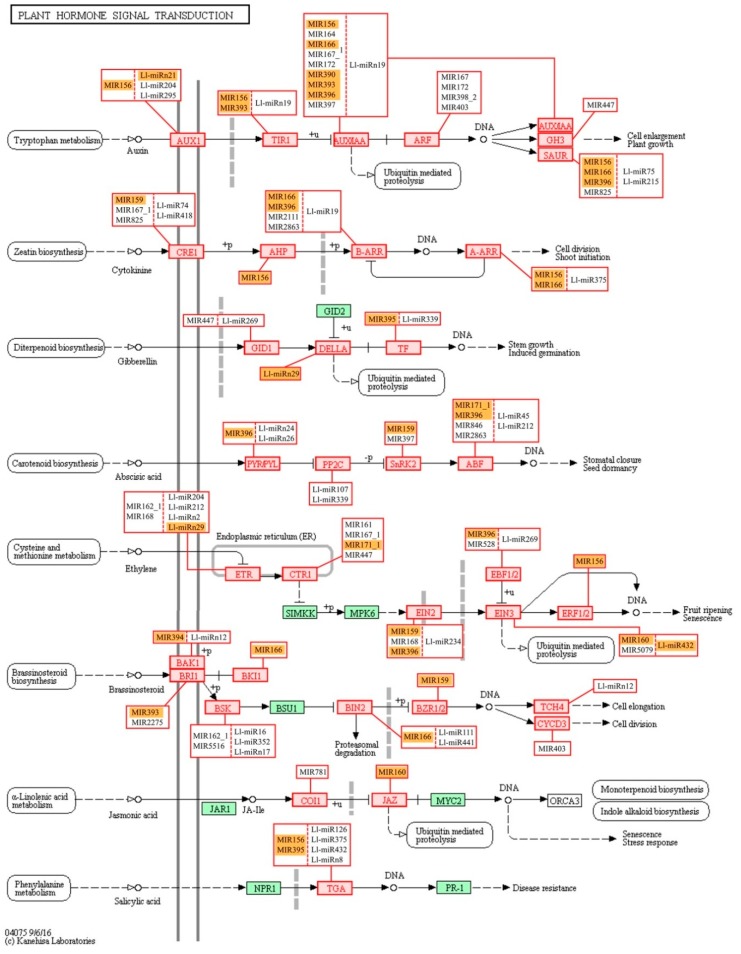
KEGG pathways related to plant hormone signal transduction targeted by known and novel miRNAs. Orange indicates DE miRNAs.

Our miRNAs targets analysis against the Kyoto Encyclopedia of Genes and Genomes (KEGG) revealed that most of the sequences in the main KEGG categories belonged to Metabolism (15,856), followed by Genetic information processing (5,267), Environmental information processing (1,517), Cellular processes (1,326) and Organismal Systems (800) (Figure S5). A full list of KEGG pathways and numbers of assigned sequences is shown in (Figures S6 and S7). One of the most represented sub-categories was Signal transduction (1,484), with over 700 putative targets in the Plant Hormone Signal Transduction pathway, where almost every sequence was frequently targeted with multiple miRNAs (Figure 10). The second most notable pathway was mitogen-activated protein kinase (MAPK) signaling, which was associated with different abiotic and biotic stress factors, with 350 putative targets distributed across every described stress response (Figure S8). A complete dataset on the KEGG analysis can be seen in (Table S16).

## 3. Discussion

Yellow lupine has great potential to become one of the leading legumes in Europe in both animal and human nutrition. Reduction the economic drawbacks resulting from excessive flower abscission would be the most convincing argument for lupine cultivation. However, this can only be achieved if we gain a deeper understanding of the plant’s biology and insight into the molecular basis for the development and maintenance of lupine flowers. Therefore, we believe that the pathways controlling these processes deserve intensive research focus. Our previous analyses of yellow lupine transcriptomes resulted in the identification of transcripts of many genes involved in flower and pod abscission and suggested sRNA involvement in this process [17]. Notably, our observations of *L. lupinus* floral development indicate that their fate (abscission or pod formation) is determined prior to AZ activation. Therefore, we decided to perform comparative analyses between sRNAs from flowers developing on the upper and lower parts of the raceme. Identifying the miRNAs and their target genes involved in the above-mentioned processes will further our knowledge of the biology of not only lupines but plants in general since the role played by sRNA in organ abscission is still obscure.

Our sRNA-seq analyses shed more light on the molecular mechanisms that control flower development of *L. luteus* and confirmed the involvement of known miRNAs, such as miR159, miR167 or miR172, in this process [60], but we have also explored the roles of sRNAs in flower abscission and identified species-specific miRNAs.

### 3.1. Known sRNAs and Their Target Genes Are Involved in Regulating Flower Development in Yellow Lupine

Among the known and conserved miRNAs a number of miRNAs commonly associated with flower morphogenesis and development, belonging to, *inter alia*, the MIR156/157, MIR159, MIR165/166, MIR167 and MIR172 families [10] were spotted.

Studies have shown that miR156 is necessary for maintaining anther fertility in *Arabidopsis*, by orchestrating the development of primary tapetum cells and primary sporogenous cells [61]. In *A. thaliana*, *SPL13B* expression is strictly limited by miR156 to anther tapetum in young buds, while *SPL2* is weakly expressed in parietal and sporogenous cells and the surrounding cell layers in young flowers [61], where it is targeted by miR156 to regulate pollen maturation [62]. MiR159 was shown to target the conserved *GAMYB-like* genes that are a part of the GA signaling pathway [63,64]. In *A. thaliana* miR159 regulates the morphogenesis of the stamen, and male fertility [65]. Two transcription factors involved in pistil and stamen development in various plant species, *ARF6* and *ARF8*, contain the target site for miR167 [66,67,68]. For *Arabidopsis*, it has been proven that both these genes are involved in stamen filament elongation, anther dehiscence, stamen maturation and anthesis [69]. In tomato, a reduction in the accumulation of the miR167-targeted *ARF6* and *ARF8* leads to the lack of trichomes on the style surface, failed pollen germination and, consequently, sterility [11]. Recent research into multiple plant species has shown that miR172 targets genes belonging to the *APETALA2* (*AP2*, *TOE1*, *TOE2*, *TOE3*) family. MiR172 is part of the photoperiodic flower induction pathway and is associated with the functioning of the ABCDE model of floral development [70]. Overexpression of *MIR172* causes formation of a phenotype characterized by the absence of perianth, transformation of sepals into pistils and early flowering [70].

Our study showed the presence of at least one member of all these families in flowers (Figure 3, Table S5), which indicated that in lupine how crucial the families were for generative development in lupine, as well. MIR156 and MIR159 are the most numerous families in *L. luteus*, which suggests they play fundamental roles in its flower development processes.

The differentially expressed miRNAs identified in yellow lupine flowers were clustered by the dynamics of their expression (Figure 5). The first cluster comprised miRNAs, the accumulation of which increased as the flowers developed, and contained miRNAs belonging to the MIR166, MIR167, MIR319, MIR390, and MIR395 families. The first of these families include Ll-miR177, which guides the cleavage of *RADIALIS*, a transcription factor from the MYB family that controls the asymmetric flower shape in *Antirrhinum majus* [71,72], as well as Ll-miR258 and Ll-miR265, which probably target the Homeobox-leucine zipper protein ATHB-15. In *Arabidopsis*, both miR165 and miR166 target the same *HD-ZIP III* genes: *ATHB15*, *ATHB8*, *REVOLUTA (REV)*, *PHABULOSA* (*PHB*), and *PHAVOLUTA* (*PHV*) to regulate gynoecium and microspore development [28,73]. In lupine the MIR167 family members that accumulate in larger quantities during flower development are Ll-miR280, Ll-miR281, and Ll-miR285, which probably target *ARF6* and *ARF8*. Ll-miR445 and Ll-miR130 are members of the MIR319 family, while their putative target genes are *TCP4* and *MYB33*, respectively. In *Arabidopsis*, the miR319a/TCP4 regulatory module is necessary for petal growth and development. Moreover, the overexpression of *MIR319* reduces male fertility, and this defect is hypothesized to be caused by the cross-regulation of *MYB33* and *MYB65* by miR319 and miR159. As the miR319 target site within the *MYB33* and *MYB65* transcripts exhibit a lower match with miRNA than the miR159 target site, the latter is more efficient at regulating these genes and miR319 is their secondary regulator [74]. This regulatory network is even more complex. In *A. thaliana*, cooperation of three miRNAs and their target genes, namely miR159/*MYB*, miR167/*ARF6/ARF8,* and miR319/*TCP4*, is a prerequisite for proper sepal, petal and anther development, and maturation. miR159 and miR319 influence the expression of *MIR167* genes, which in turn affect each other. These miRNAs orchestrate plant development by regulating the activity of the phytohormones GA, JA, and auxin [75]. Increased accumulation of miR167 and miR319 in the late stages of yellow lupine flower development could also be associated with regulating the growth and development of petals and anthers. Another miRNA showing a similar expression profile is Ll-miR9/miR390-5p. In lupine, it targets the *TAS3* transcript, which in turn is a source of tasiR-ARF, a negative regulator of *ARF2*, *ARF3* and *ARF4* activity. This regulatory cascade plays a vivid role in development of many plant species [76]. The expression level of miR390 derived from *MIR390b* reflects auxin concentration in organs, while the repression of *ARF2*, *ARF3,* and *ARF4* by tasiR-ARF are important for lateral organ development [18,77], and flower formation [78]. Ll-miR118 and Ll-miR119, which target ATP sulfurylase (*ATPS*) according to our degradome data, belong to the MIR395 family. In *Arabidopsis*, miR395 targets two gene families, ATP sulfurylases and sulfate transporter 2:1 (*SULTR2:1*), which are elements of the sulfate metabolism pathway [79]. ATPS regulates glutathione synthesis and is an essential enzyme in the sulfur-assimilatory pathway [80]. In cotton, the miR395-APS1 module is engaged in drought and salt stress response [81]. Sulfate is the main source of sulfur and is taken up by roots, transported throughout the plant and used for assimilation. Sulfate limitation forces a significant up-regulation of miR395 expression [82]. Presumably, during yellow lupine flower development, the demand for sulfur increases and the plant activates mechanisms for its efficient uptake.

Within the cluster of miRNAs, the expression of which decreased as the flowers developed, there were homologues of miR390-3p, miR858, miR396-3p, miR168, miR408-3p and miR398 (Figure 5). Ll-miR99, Ll-miR100, and Ll-miR102 are identical to miR390-3p (the so-called passenger strand, former star strand). However, their expression showed an opposite trend to that of miR390-5p. The differential expression and functioning of passenger miRNAs have already been described. The research carried out by Xie and Zhang in 2015 on cotton showed that the formation of some miRNA*s, such as miR172* and miR390*, was associated with the phases of the plant’s growth [83]. Therefore, miRNA*s can be specifically expressed in various tissues to maintain the steady state of the organism. Our degradome analysis for yellow lupine showed that Ll-miR9/miR390-5p was able to guide the cleavage of the *TAS3* transcript. There is no certainty as to the status of its passenger strand, which suggests its locally limited activity or its involvement in regulation of other targets and further research is required to identify its accumulation and function in the organs concerned. Another miRNA from the cluster is Ll-miR155/miR396-3p (passenger strand), which guides cleavage of JMJ25 demetyhylase mRNA (confirmed in degradomes), involved in preserving the active chromatin state [84]. *ECERIFERUM1* (*CER1*), the target gene in lupine for another two homologues of miR396-3p, Ll-miR199 and Ll-miR200, is a homologue encoding an enzyme involved in alkane biosynthesis, and in cucumber is engaged both in wax synthesis and ensuring pollen viability [85]. This cluster also included a miRNA that negatively regulates elements involved in miRNA and ta-siRNA functioning, namely Ll-miR247/miR168 targeting *AGO1* mRNA [86]. Another miRNA clustered here was the highly conserved Ll-miR60/miR408-3p, which guides the processing of the mRNA of the copper-binding Basic Blue protein homologue (plantacyanin, PC). In *Arabidopsis*, PC plays a role in fertility, exhibiting the highest expression in the inflorescence, especially in the transmitting tract. [87]. Transgenic *Arabidopsis* plants over-expressing *MIR408* displayed altered morphology, including significantly enlarged organs, resulting in enhanced biomass and seed yield. Plant enlargement was shown to be primarily caused by cell expansion rather than cell proliferation, and in transgenic plants it was correlated with stronger accumulation of the myosin-encoding transcript and gibberellic acid [88]. It seems that high expression levels of miRNAs grouped in the cluster are correlated with intensive growth and differentiation of young floral tissues.

Among the miRNAs identified in yellow lupine several that seemed to be crucial in particular stages of the plant’s development were spotted (Figure 4, Table 4, Table S7). For example, the largest quantities of miR159 (Ll-miR452 and Ll-miR454) were accumulated in stages 2 and 3 of the plant’s development. According to degradome data they targeted *GGP-5* (*GAMMA-GLUTAMYL PEPTIDASE 5*) of an undefined function in plants, and an evolutionarily conserved target for *GAMYB*, respectively. As already mentioned, this could be associated with miRNA family cooperating with miR167 and miR319 in regulating *L. luteus* anther maturation. The accumulation of Ll-miR251/miR5168-3p, Ll-miR92/miR1861b, Ll-miR229/miR369-5p, and Ll-miR311/miR5794 increased in stage 2 upper and lower flowers, while – interestingly – in the later stages these miRNAs were only present in lower flowers. According to degradome analysis, Ll-miR251/miR5168 guides cleavage of the mRNAs of the genes encoding the Homeobox-leucine zipper protein ATHB-14 and the chaperone protein dnaJ 13. The miR5168 sequence displays a great similarity to that of miR166, thanks to which they may perhaps share the same target gene *ATHB-14*, the putative transcription factor engaged in the adaxial-abaxial polarity determination in the ovule primordium in *A. thaliana* [89]. As confirmed by yellow lupine degradome sequencing, Ll-miR229/miR396-5p targets *GROWTH-REGULATING FACTOR 5* (*GRF5*) and *GRF4* transcripts. In *Arabidopsis*, *GRF5* is expressed in anthers at early stages of flower development and in gynoecia throughout the whole flower development, and transcripts of *GRF4* accumulate later in sepals, tapetum, and endocarpic tissues of ovary valves [90]. Transgenic rice with Os-miR396 overexpression and *GRF6* knock-down suffers from open husks and sterile seeds [91]. *GRF6* cooperates with *GRF10* to transactivate the *JMJC* gene *706* (*OsJMJ706*) and *CRINKLY4 RECEPTOR-LIKE KINASE* (*OsCR4*) responding to GA, which is a prerequisite for the flower to successfully develop into a normal seed [91]. An increased share of miRNAs involved in cell division, namely miR396, miR319, and miR164, in NGS analyses was also observed in early grain development in wheat [92].The presence of these miRNAs in yellow lupine flowers suggests that their regulation of cell proliferation also plays an important role in development of generative organs.

### 3.2. Involvement of New miRNAs in L. luteus Flower Development

Using ShortStack [53] software we predicted 28 candidates for new miRNAs (Table 3). Interestingly, many of these novel miRNAs showed similarity to precursor miRNAs from miRBase, which leads to the conclusion that they might be new members of the already known families, for example MIR167 (Ll-miRn12 and Ll-miRn27), MIR172 (Ll-miRn4), MIR393 (Ll-miRn19) or MIR169 (Ll-miRn3, Ll-miRn11, and Ll-miRn15) (Table S6).The other 13 had no homologues among known miRNAs and were recognized as lupine-specific miRNAs. Some of the new miRNAs displayed differential expression during *L. luteus* flower development. Ll-miRn3, which shows similarity to pre-miR169, displayed differential expression in UF1 vs LF1 and LF2 vs LF1 library comparisons, wherein it is the most accumulated in LF1, and in flower pedicels (up-regulated in FPNAB). According to degradome data, this miRNA targets *SCARECROW2* (*SCR2*) homologue, a putative activator of the calcium-dependent activation of *RBOHF* that enhances reactive oxygen species (ROS) production and may be involved in cold stress response [93]. In rice *SCR2* expression is relatively high in flower buds and flowers, and after flowering rises in the leaves and roots [94]. In yellow lupine, this gene may be involved in intense cell divisions during early flower development and is down-regulated in the pedicels with an active AZ to stop its growth. Another frequently encountered novel DEmiR was Ll-miRn22, which shows sequence similarity to pre-miR1507, is up-regulated in LF3 vs LF2 and LF2 vs LF1 library comparisons, and its expression escalates with flower development in the bottom whorl. The MiR1507 family is annotated as legume-specific [95]. Through analyses of our degradome data we have not found its target gene, and the psRNATarget hit was the putative disease resistance RPP13-like protein 1. Unfortunately, this protein has been poorly described, therefore it is difficult to determine its function in yellow lupine flowers. Noteworthily, the target genes of Ll-miRn1 and Ll-miRn30 identified through degradome sequencing are *SGS3* and *DCL2*, respectively, and the miRNAs are up-regulated in LF3 vs LF2 comparisons and down-regulated in UF1 vs LF1 comparisons, respectively. *SGS3-* and *DCL2*-encoded proteins are involved in sRNA biogenesis [96]. Importantly, novel miRNA identified in soybean Soy_25 displays high sequence similarity to Ll-miRn1 and also targets *SGS3*, which indicates that this regulatory feedback loop for sRNA biogenesis is common for *Fabaceae* [97]. These results indicate that *L. luteus* miRNAs play a regulatory role in siRNA biogenesis in early flower development.

### 3.3. miRNA Accumulation Varies in Lower and Upper Flowers in Different Stages of Development

One of our goals was to identify the sRNAs engaged in yellow lupine flower development, with a particular emphasis on the differences between flowers from lower and upper parts of the inflorescence, in order to gain an insight into how early the flower fate is determined.

In our study, we spotted differences in miRNA accumulation patterns as early as the first stage of flower development.

Flowers collected from the lower whorls displayed higher accumulations of sequences corresponding to miR5490, miR5794, miR1861, miR396-5p, miR395, miR166, and miR159-3p (Table 5). miR1861 and miR396 were recognized as positive cell proliferation and development regulators [98,99,100]. In rice, for example, miR1861 exhibited differential expression during grain filling [101], and its expression was higher in superior grains in comparison to inferior ones [102]. This is consistent with our hypothesis, that a higher occurrence of miR1861 and miR396 in lower flowers may be an indication of the plant investing more supplies in this part of the inflorescence.

From the second stage until the end of their development, upper flowers accumulated more miRNAs corresponding to miR319, miR394, miR160, and miR393 (Figure 4, Table 5). MiR393 regulates the accumulation of transcripts encoding auxin receptors belonging to the TAAR family. Changes in receptor abundance affect the sensitivity of the given tissue to auxin and this is how this molecule influences plant development [102]. In *A. thaliana*, miR160 directly controls three *ARF* genes, namely: *ARF10, ARF16* and *ARF17* [103]. In tomato, sly-miR160 is abundant in ovaries, and changes in its expression affect plant fertility [12]. Down-regulation of sly-miR160 caused improper ovary patterning and thinning of the placenta already prior to anthesis [12]. In view of these facts, higher expression of miR160 in lupine upper flowers in their development means that a slightly different organization of the gynoecia may be one of the crucial determinants of flower fate. Additionally, the elevated expression levels of miR160 and miR393 in upper flowers of lupine suggest a reduction in the abundance of the transcripts of their target genes encoding auxin receptors and auxin response factors. This, in turn, may have led to a reduction in auxin sensitivity. Decreasing the number of transcription factors belonging to the TCP family (targeted by miR319), probably caused different cell proliferation profiles in flowers collected from the upper whorls.

Additional expression studies of selected miRNA (Ll-miR281/miR167, Ll-miR224/miR393, Ll-miR333/miR160, Ll-miR329/miR160) carried out in the upper flowers of yellow lupin developing after removal of the lower ones (UFR) (Figure S2), and consequently with a changed, when compared to the original, fate, provide additional confirmation of the results obtained from RNA-seq analysis (Figure S3).

### 3.4. sRNAs Are Involved in Flower Abscission in L. luteus

Little is known about sRNA engagement in flower abscission. Research on the involvement of miRNAs in this process has been already carried out in cotton [104], tomato [12,105], and sugarcane [106]. For a genome-wide investigation of miRNAs involved in the formation of the abscission layer in cotton, two sRNA libraries were constructed using the abscission zones (AZ) of cotton pedicels treated with ethephon or water. Among the 460 identified miRNAs, only gra-MIR530b and seven novels showed differential expression in abscission tissues [104], and these miRNAs have no homologues in our dataset.

Besides ovary patterning in tomato, sly–miR160 regulates other two auxin-mediated developmental processes: floral organ abscission and lateral organ lamina outgrowth [12]. In that study, down-regulation of sly-miR160 and the resulting higher expression of its target genes, transcriptional repressors of auxin response *ARF10* and *ARF17*, also resulted in the narrowing of leaves, sepals and petals and an impeded shedding of the perianth after successful pollination [12]. This was consistent with the higher accumulation of Ll-miR329/miR160-5p, Ll-miR332/miR160-5p, and Ll-miR333/miR160-5p in upper flowers designated to fall off in yellow lupine. As these miRNAs showed no differential expression in flower pedicels, it probably does not play a role in the executory module of abscission itself but is rather a part of a mechanism that determines flower fate.

Another research on tomato using sRNA and degradome sequencing libraries explored the roles of sRNAs in AZ formation in the early and late stages of the process additionally accelerated or not by ethylene or control treatment [107]. The study showed that in tomato pedicels, the accumulation levels of, *inter alia*, miR156, miR166, miR167, miR169, miR171, and miR172 rose in late stages of abscission, while the abundance of miR160, miR396 and miR477 dropped [107]. Although it is difficult to compare ethylene-treated tomato pedicel results to our data, it is worth noting that in the corresponding FPAB vs. FPNAB comparison in our study, the accumulation of some miRNAs was similar: miR396 level was lower, and the levels of miRNAs annotated as miR167 and miR166 were higher in FPAB (Table 6).

It has been proven for sugarcane that among others both mature (5p) and passenger (3p) miRNAs from MIR167 family were up-regulated in ‘leaf abscission sugarcane plants’ comparing to ‘leaf packaging sugarcane plants’ (which corresponds to the FPAB vs. FPNAB comparison in our study) [106]. In our study, both mature and passenger members of the MIR167 family were leaders among DEmiRs, too, (Table 6) pointing to their crucial role in both vegetative and generative organ abscission. Significantly, this applies to evolutionarily distant taxa: both monocots and dicots.

In our paper, among the up-regulated miRNAs, the most numerous family besides already mentioned MIR167 was MIR398 with 3 members being among top-regulated ones. Among the down-regulated miRNAs, the members of MIR390, MIR396 and MIR395 families were most abundant. It was shown for other plant species, that these miRNAs are engaged in the regulation of auxin signal transduction pathway (miR167 and miR390 [108]), regulation of cell division (miR396 [100]) and stress response (miR395 [81,82]).

It is worth noting, that in comparisons of *Lupinus* pedicel libraries there are novel miRNAs: three are down-regulated in FPAB and one is up-regulated. Furthermore, Ll-miRn3 is up-regulated in both, young flowers designated to be maintained on the plant (LF1) and pedicels with inactive AZ (Table 6), which may indicate its role in preventing flower abscission. In the future, it is worth examining the role of its target gene, which encodes a protein that does not resemble any known protein.

With regard to siRNAs, the most up-regulated ones in FPNAB were: Ll-siR173, Ll-siR4 and Ll-siR13, and the most down-regulated one were Ll-siR208. Unfortunately, the lack of literature data on their targets makes it impossible for the specifics of their function in the studied process. However, it is worth mentioning, that in pedicels high levels of accumulation are displayed by siR249/tasiR-ARF and siR308/tasiR-ARF, which target transcripts encoding ARF2, ARF3 (confirmed in degradomes). These results strongly suggest the involvement of siRNAs in the functioning of lupine pedicels.

### 3.5. Possible miRNA-dependent Regulatory Pathways That Participate in Development and Abscission of Yellow Lupine Flowers

Recent studies have shown that sRNA activity is associated with the hormonal regulation of plant development through influencing the spatio-temporal localization of the hormone response pathway [109].

The auxin signal transduction pathway mainly consists of three elements. Auxin is perceived by members of the TAAR family. There are AUX/IAA repressor proteins and ARF transcription factors downstream of these receptors [110,111,112]. The expression of *TAAR* receptors is regulated by miR393 and secondary ta-siRNA derived from their own transcripts [20]. miR167 and miR160 affect the *ARF6*, *ARF8* [67] *ARF10*, *ARF16* and *ARF17* [113] transcript accumulation, respectively. It has been proven that the expression of *ARF2*, together with *ARF3* and *ARF4*, is regulated by the ta-siRNA/miR390 module [114]. In the two-hit model, ta-siRNA-containing the *TAS* transcript is recognized by two miR390 molecules, one of which guides its cleavage, and the other, in a complex with AGO7, serves as a primer for complementary strand synthesis, with its subsequent processing ultimately resulting in ARF-targeting siRNA biogenesis [115].

In our study, among the differentially expressed sRNAa in flowers and flower pedicels, there were members of the MIR167, MIR160, MIR393 and MIR390 families, as well as phased siRNAs targeting *ARF2*, *ARF3*, and *ARF4*. This fact suggests a vivid role of auxin-related sRNAs in flower development and abscission in *L. luteus* and confirms our previously published results of transcriptome-wide analyses, where we observed differences in expression levels of genes encoding several elements of the auxin signal transduction pathway [17]. The relatively high number of members of the MIR167 family showing differential expression in the studied variants indicates that miR167 is one of the key regulators of flower development and abscission in yellow lupine.

*Lupinus LlARF2*, *LlARF3,* and *LlARF4* transcripts are possibly down-regulated in the processing that is guided by Ll-siR249 and Ll-siR308 (Table 4), which are identical to tasiR-ARFs in many plant species according to the tasiRNAdb database [116]. These tasiR-ARFs probably originate from *TAS3* transcript (TRINITY_DN55534_c4_g1) containing two binding sites for miR390 (Figure S9a). Ll-miR9/miR390and surprisingly also Ll-siR240, guide the cleavage of another *TAS3* mRNA (TRINITY_DN54998_c6_g5_i2) (Figure S10) which contains only one target site for miR390 (Figure S9b). This is the first report on *TAS3* processing regulated by siRNA. The target site for Ll-siR240 is shifted by 10 nucleotides relative to the target site for Ll-miR9/miR390 (Figure S10). The expression of Ll-siR249, Ll-siR308, and Ll-miR9 showed a similar profile, as it rose during flower development and was the highest in the pedicels (Figure 7). Ll-siR240 accumulated proportionally to *TAS3* with only one target site for miR390, which means that it was least expressed in the pedicels, while in flowers its expression increased with time (Table S18). The identified target transcripts belonging to the *ARF2*, *ARF3,* and *ARF4* gene families showed differential expression but with no clear trend (Table S18). This may indicate that these siRNAs act locally, repressing only a pool of transcripts expressed in a given tissue, while in other flower parts activity of these genes is regulated in other ways. The presence of all the elements of the miR390/TAS3/tasiR-ARF module among the DE sRNAs in yellow lupine suggests that alterations in its functioning have a great impact on *L. luteus* flower development. The additional element in the form of siRNA that processes *TAS3* mRNA seems to be a new species-specific adjuster of this regulation module.

We have also performed GO enrichment analysis of the target genes for sRNAs identified in flowers of yellow lupine (Figure 9, Figure S4a,b, Table S10). What is most interesting is that quite a considerable number of target genes fell within the ‘response to stimulus’ and ‘signaling’ categories, which means that miRNAs modulated the way the plant adapted to environmental stimuli (Figure 9). An in-depth analysis of GO terms concerning plant hormones (Figure S4a) showed that most of the miRNAs identified in yellow lupine modulated more than one hormone signaling pathway. For example, Ll-miR181 belonging to the MIR166 family modulated processes associated with four hormones, namely auxin, gibberellin, jasmonic acid, and salicylic acid, by targeting not only transcription factor AS1, a central cell division regulator [117], but also Cullin-3A, an element of the ubiquitination complex [118]. Another two members of this family, Ll-miR173 and Ll-miR177, targeted the same gene, *26S PROTEASOME NON-ATPASE REGULATORY SUBUNIT 8 HOMOLOG A* (*RPN12A*), involved in the ATP-dependent degradation of ubiquitinated proteins during auxin and cytokinin response [119]. Our GO analysis for yellow lupine flowers additionally showed that miRNAs were responsible for guiding the processing of genes simultaneously involved in multiple processes associated with flower development (Figure S4b). For example, in many plants *AP2* is involved in the specification of floral organ identity [120], as well as ovule [121] and seed development [122,123], and in our study, it was targeted by ten lupine miRNAs. On the other hand, seven of these miRNAs additionally targeted a homologue of negative flower development regulator, *LIKE HETEROCHROMATIN PROTEIN 1* (*LHP1*) [124]. This highly degenerated and ambiguous model of gene regulation by lupine miRNAs shows that in this plant the adjustment of key biological processes related to fertility is a complex network of interconnected factors.

We have also conducted KEGG functional analysis of the putative targets identified for miRNAs in lupine which indicated their engagement in regulating a number of metabolic pathways—especially ‘carbohydrate metabolism’ and ‘nucleotide metabolism’ (Figure S5). ‘Carbohydrate metabolism’ was also one of the most enriched KEGG pathways in our previous *L. luteus* transcriptome analysis [17], and its activation may be an indication of cell walls being rebuilt or changes in nutrient supply. The next most numerous group of miRNA targets was categorized into the ‘Genetic information processing’ KEGG pathways, namely, ‘spliceosome’, ‘RNA transport’, and ‘ubiquitin proteolysis’. This suggests that in yellow lupine flowers most miRNAs regulate processes related to post-transcriptional events and protein degradation. Three KEGG categories within the ‘Environmental information processing’ category is extremely important in terms of plant development, and they are ‘Signal transduction pathways’ comprising the MAPK cascade, ‘phosphatidylinositol’ and ‘plant hormone’ signaling pathways (Figure 10, Figure S6, S7, S8). The MAPK pathway is involved in regulating several processes, such as biotic and abiotic stress response (reviewed in [125,126]), and associated with the functioning of hormones such as ethylene [127] and abscisic acid, engaged in organ abscission and other processes (reviewed in [128,129]). The MAPK cascade is also an element of the positive feedback loop amplifying the abscission signal [130]. Auxin seems to be major target of sRNAs in yellow lupine. However, KEGG enrichment analyses of the identified target genes for lupine miRNAs indicated that the signal transduction pathways of gibberellin, cytokinin, the already mentioned ethylene, and ABA were potentially modulated by miRNAs in *L. luteus*, as well, but in less extent (Figure 10).

Interestingly, like in the case of GO analysis, KEGG analysis for the MIR166 family showed that it was involved in the auxin, cytokinin, and brassinosteroid signal transduction pathways (Figure 10). These data show again how the fine-tuning of expression of phytohormone-related genes by sRNAs is important for growth and development regulation.

## 4. Materials and Methods

### 4.1. Plant Material

Commercially available seeds of yellow lupine cv. Taper were obtained from the Breeding Station Wiatrowo (Poznań Plant Breeders LTD. Tulce, Poland). Seeds were treated with 3,5ml/kg Vitavax 200FS solution (Chemtura AgroSolutions, Middlebury, United States) to prevent fungal infections and inoculated with cultures of *Bradyrhizobium lupine* contained in Nitragina (BIOFOOD s.c., Walcz, Poland) according to seed producer’s recommendations [131]. All the research material used for RNA isolation was collected from field-grown plants cultivated in the Nicolaus Copernicus University’s experimental field (in the area of the Centre for Astronomy, Piwnice near Torun, Poland, 53°05’42.0”N 18°33’24.6”E) according to producer’s agricultural recommendations [131] until the time of flowering. Flowers were collected 50 to 54 days after germination from the top and bottom parts of the inflorescence and were separated into four categories based on the progression of their development. Flower pedicels from flowers undergoing abscission or maintained on the plant were also collected, as in our previous study [17].

Plants with the same number of flower whorls were selected for the flower removal experiment and control. All plants were grown as described above up to the flowering stage. When plants reached the stage at which the top-most flowers were in the developmental stage S1, other flowers were removed from the inflorescence (UFR samples). The samples were collected for the gene expression analysis in the stages S1–S4. As a control, flowers from stages S1–S4 from upper (UF) and lower (LF) part of the inflorescence were collected.

### 4.2. RNA isolation, Library Construction, and RNA Sequencing

Total RNA from at least 5 plants (25 flowers or pedicels) for each variant was performed using the miRNeasy Mini Kit (Qiagen, Venlo, the Netherlands) including on-column DNA digestion with the RNase-Free DNase Set (Qiagen, Venlo, the Netherlands). The total RNA quality and quantity were evaluated with agarose gel electrophoresis and Nanodrop ND-1000 spectrophotometer (Thermo Scientific Waltham, MA, USA). Both the RNA Integrity Number (RIN), and RNA concentration were measured with the 2100 Bioanalyzer (Agilent Santa Clara, CA, USA) using the Small RNA Kit (Agilent Santa Clara, CA, USA). All the samples had adequate concentrations of RNA and RIN ranging from 8.9 to 10.0 and were sent for library construction to Genomed S.A (Warszawa, Poland) and sequencing BGI (Shenzhen, China).

Small RNA libraries were prepared from the total RNA using the NEBNext Multiplex Small RNA Library Prep kit for Illumina (New England Biolabs, Ipswich, MA, USA) and subsequently sequenced on the HiSeq4000 platform (Illumina, San Diego, CA, USA) in the 50 single-end mode. All libraries were constructed in two biological replications resulting in a total number of 20 sRNA libraries.

The total RNA extracted from pooled material derived from three biological replicates was used to prepare ten transcript libraries using the NEBNext Ultra Directional RNA Library Prep Kit for Illumina (New England Biolabs, Ipswich, MA, USA) and sequenced on the HiSeq4000 platform in the 100 paired-end mode.

For degradome sequencing, total RNA from three biological samples of UF3 and LF3 was pooled to maximize the amount of required material. The protocol for degradome library preparation comprised the following steps: (i) mRNA isolation, where poly (A)-containing mRNA molecules are purified from total RNA using poly(dT)oligo-attached magnetic beads, (ii) synthesis and subjugation of cDNA to ligate 5′ adaptors, and purification of the resulting products with TAE-agarose gel electrophoresis, (iii) PCR amplification to enrich the final products, (iv) library-quality validation on the Agilent Technologies 2100 Bio-analyzer and using the ABI StepOnePlus Real-Time PCR System (Applied Biosystems, Foster City, CA, USA), and (v) sequencing of the prepared library on the HiSeq4000 platform in the 50 single-end mode.

### 4.3. De Novo Transcriptome Assembly and Gene Expression Analysis

The transcriptome was assembled *de novo* from RNA-Seq data using Trinity v 2.4.0 (https://github.com/trinityrnaseq/trinityrnaseq/releases) with default settings as in our previous study [17]. The expression level was estimated at both the unigene and isoform levels and described by FPKM (Fragments Per Kilobase Of Exon Per Million Fragments Mapped): the number of reads per unigene normalized to the library size and transcript length using RSEM [132] as previously described [17].

### 4.4. Identification of Known and Potentially Novel miRNAs and Phased siRNA

Adapter-free sRNA reads were subjected to quality filtering with fastq_quality_filter from the FASTX-Toolkit package (http://hannonlab.cshl.edu/fastx_toolkit/) using -p 95 and -q 20 parameters (http://hannonlab.cshl.edu/fastx_toolkit/commandline.html#fastq_quality_filter_usage). Then, redundant and counting read occurrences (i.e., raw expression values) were identified with the fastx_collapser from the same package.

Short reads were compared against noncoding RNAs from Rfam [49,50] and both mature miRNAs and their precursors from miRBase [51]. The comparison was performed with Bowtie [133] allowing for no mismatches.

To identify phylogenetically conserved mature miRNAs with sequences and lengths identical to known plant miRNAs we searched miRBase for similarity at the mature miRNA level.

To predict potential novel miRNAs we applied ShortStack [53] with default settings. This tool identifies miRNAs based on their mapping against a reference genome. Since no genome was available for the studied species, we used de novo approach for transcriptome assembly instead. The latter method allowed for identification of miRNAs that showed no similarity to miRNAs annotated in miRBase and these miRNAs were assigned as new.

ShortStack [53] was used to identify small RNAs that were being cut in phase from longer precursors (phased siRNAs) with transcriptomes used as references. The top 200 candidates were selected from each sample, based on the phased score value provided by ShortStack. Finally, lists of such sRNAs from all samples were merged into a single dataset of non-redundant phased siRNAs (Table S6). The expression values were calculated as in the case of miRNAs.

### 4.5. Small RNA Expression Quantifications and Analysis of Differentially Expressed si- and miRNAs

MiRNA counts within each sample were first normalized to RPM values (reads per million values) and then a differential expression analysis was performed with the DESeq2 R package [134]. The files containing raw read counts for miRNAs/siRNAs from treatment and control replicates were used as input, and only candidates with an adjusted *p*-value (*q*-value) below 0.05 were retained for further analysis.

### 4.6. Identification of sRNA Targets

For target prediction using degradome analyses after sequencing, the reads were filtered using fastq_quality_filter from the Fastx-Toolkit package (http://hannonlab.cshl.edu/fastx_toolkit/) with at least 95% of nucleotides in each read demonstrating quality >= 20 (Phred Quality Score) with -p 95 and -q 20. The filtered Degradome-seq data, sequences of mature miRNA/siRNA and the assembled transcriptomes were processed with the CleaveLand4 package [58] to determine the cleavage sites for sRNA using default program settings. The final results were filtered based on the *p*-value < 0.05.

To predict targets for known or novel miRNAs, and phased siRNAs, we used also the psRNATarget tool [59] querying the assembled transcriptomes with the default Schema V2 (2017 release) and an expectation score of up to 4.

### 4.7. Evolutionary Conservation of miRNAs

*L. luteus* miRNAs were assigned to miRNA families based on miRBase classification, and the same was done for the sequences of all *Eudicotyledons* species present in miRBase, with the exclusion of *Gossypium arboretum* (which has only one sequence deposited in the database that cannot be classified as belonging to any known miRNA family). miRNAs from 52 species were compared against *L. luteus* miRNAs in order to count the numbers of miRNA family members shared amongst the species. The same analysis was performed with data narrowing to nine *Fabaceae* species.

### 5.8. Expression analysis with RT-qPCR

MiRNAs and siRNAs expression analysis was performed using the Stem Loop RT-qPCR technique, according to [56] with some modifications. An RT primer specific for each sRNA was used for the reverse transcription using total RNA of each sample and the SuperScript III Reverse Transcriptase (Thermo Fisher Scientific, Waltham, MA, USA) in a 20 µL reaction volume. To increase the accuracy and efficiency of the reaction, the pulse RT approach [57] was applied to the reverse transcription which consisted of two steps: 30 min of pre-incubation at 16 °C, followed by 60 cycles at 30 °C for 30 s, 42 °C for 30s and 50 °C for 1 s. qPCR was subsequently performed using specific primers designed according to [57], modified so that the UPL9 hydrolysis probe (Roche, Basel, Switzerland) was used for maximization of accuracy and background reduction. This reaction was performed using the SensiFAST Probe No-ROX kit (Bioline meridian bioscience Cincinnati, OH, USA) and the LightCycler 480 (Roche, Basel, Switzerland). Each 20 µL reaction contained: 1 µL cDNA template (transcribed from ~100 ng of total RNA for less expressed miRNAs and 25 ng of total RNA for more expressed miRNAs), 1 µL of 10 µM qPCR specific forward primer, 1 µL of 10 µM Universal-qPCR primer, 10 µL of 2× SensiFAST Probe No-ROX Mix, 0.2 µL of 10 µM UPL9 probe and 6.8 µL ddH_2_O. qPCR was executed by pre-incubation at 95 °C for 10 min, followed by 45 cycles of 95 °C for 10 s, 59 °C for 30 s, and 72 °C for 1 s. Target gene expression was performed as in [17]. Each experiment consisted of three biological and technical replicates. The relative expression levels were calculated using the 2^−∆∆*C*t^ method, and the data were normalized to the CT values for the *LlActin* reference gene (according to [17]). All primer sequences are given in Table S10.

### 4.9. Gene Ontology (GO) and KEGG Analysis of Target Genes

In order to estimate the potential roles of *L. luteus* sRNAs in biological processes, GO annotations of their target genes were downloaded from the Gene Ontology using NCBI or UniProt identifiers The Bioconductor GOseq package [135] was used for GO enrichment analysis. KEGG annotation and enrichment analysis were performed to determine the metabolic pathways. The GO terms and KEGG pathways were considered to be significantly enriched with the corrected *p*-value of 0.05, which was calculated using a hypergeometric test [136].

### 4.10. Data submission to Sequence Read Archive NCBI

The RNA-Seq and small RNA-Seq data have been uploaded to the SRA database and are available under BioProject ID PRJNA419564 and Submission ID SUB3230840.

## 5. Conclusions

**Figure 11:**
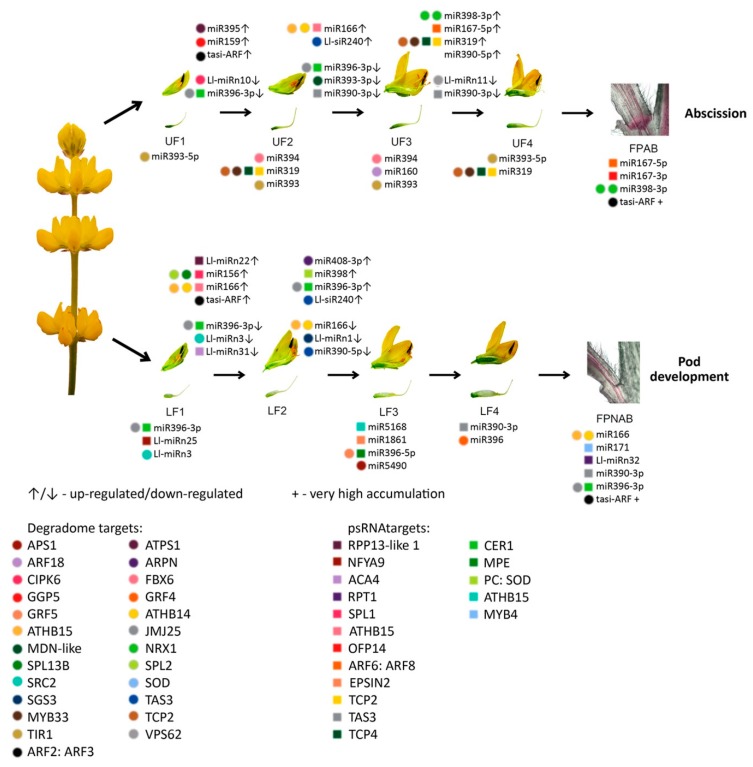
MiRNAs and siRNAs participating in yellow lupine flower development and abscission. Scheme based on the results of the current analysis. Arrows pointing upwards and downwards represent sRNAs that are up or downregulated in the transition between two developmental stages, respectively. The plus sign marks significantly expressed sRNAs. Colored circles represent targets found in the degradome, colored squares represent targets found using psRNATarget, as listed below. Multiple markers indicate that the sequence has multiple targets. Abbreviated gene names were acquired from UniProt database, where full target names can be found. Pictures from left to right are as follows: 4-whorl inflorescence of yellow lupine, flower cross-sections and isolated pistils for each stage of development, cross-sections of abscissing and non-abscissing flower pedicels stained with phloroglucinol-HCL solution.

In this paper, we present the first case of identification and integrated analysis of small ncRNA, transcriptome, and degradome sequencing data, which allowed us to identify known and novel miRNAs, siRNAs and their target genes probably involved in regulating yellow lupine flower development and abscission. These miRNAs and siRNAs, by controlling the expression of their target genes, may have an impact on the development and fate of flowers growing in particular parts of the inflorescence (Figure 11). There appear to be microRNAs controlling auxin signal transduction elements and proliferation regulators in n the central node of the regulatory network controlling flower development or abscission. In addition to the purely cognitive aspects of describing the evolutionary conservation and the species specificity of important mechanisms regulating plant development, this work may contribute to the optimization of field crops and to monitoring the impact of various factors on flowering in yellow lupine. The use of the NGS technique allows for a detailed analysis of the regulatory networks which include sRNAs and their target genes. However, the results of sRNA-seq also contain a large number of uncharacterized sRNAs, the function of which may also have significance for the studied processes. More experimental and bioinformatic research is needed to fully describe the complex mechanisms of plant development regulation by low-molecular-weight regulatory RNAs.

## Figures and Tables

**Table 1:** Summary of reads and general annotation of small RNA-seq data.

	FPAB	FPNAB	LF1	LF2	LF3	LF4	UF1	UF2	UF3	UF4
**All reads**										
unique	5,915,879.5	6,387,744.5	6,755,903	6,725,750	6,401,622.5	6,434,224.5	6,633,250	6,525,844.5	6,061,243.5	6,188,739.5
redundant	15,042,451	14,794,948	15,357,830	15,397,345	15,132,153	15,067,593	15,368,697	15,249,713	14,996,762	15,226,142
**Annotation**										
Unique										
miRBase	424	388	449	399	346	412	467	336	360	410
hairpin	2001	1832	1713	1610	1738	1801	1815	1699	1750	1995
Rfam	45,858	31,044	25,877	29,300	34,998	33,577	31,265	33,221	36,206	43,875
unknown	5,867,598	6,354,480.5	6,727,865	6,694,441.5	6,364,541.5	6,398,435	6,599,704	6,490,589.5	6,022,928	6,142,461
All										
miRBase	580,674	562,932	364,583	351,641	394,114	410,044	368,739	448,377	571,963	471,398
hairpin	298,173	319,855	208,266	234,335	286,889	274,200	192,098	236,605	298,894	301,915
Rfam	731,119	493,959	299,502	386,808	581,709	528,515	522,096	499,417	555,178	727,483
unknown	13,432,486	13,418,203	14,485,480	14,424,562	13,869,443	13,854,835	14,285,765	14,065,314	13,570,728	13,725,347

**Table 2:** Rfam annotation summary.

	FPAB	FPNAB	LF1	LF2	LF3	LF4	UF1	UF2	UF3	UF4
tRNA	4742	3467	2537	3245	3771	4209	2914	3617	4115	4645
rRNA	33,810	23,320	19,921	22,331	27,302	25,453	24,092	25,477	27,557	34,641
snoRNA	2164	893	496	561	540	591	1125	683	827	748
Intro	1480	1238	1094	1201	1403	1340	1166	1339	1407	1546
Retro	829	800	681	707	792	742	803	766	751	852
U1	415	100	64	103	83	66	62	84	124	124
U2	620	323	263	261	286	294	275	308	312	346
U3	433	244	150	172	169	163	189	184	245	215
U4	248	61	51	63	54	54	58	67	91	82
U5	69	10	12	15	16	16	13	9	13	21
U6	349	81	52	81	64	58	102	76	90	108
Total	45,858	31,044	25,877	29,300	34,998	33,577	31,265	33,221	36,206	43,875

**Table 3:** Novel miRNAs identified in *Lupinus luteus*.

miRNA ID	Sequence (5’–3’)	Size (nt)	Precursor (RNA-seq ID)	LP (nt)	MFE (kcal/mol)	Target Description (degradome/psRNAtarget)
Ll-miRn1	TTGCCAATTCCACCCATGCCTA	22	TRINITY_DN58100_c0_g3_i1	125	−59.90	**SUPPRESSOR OF GENE SILENCING 3**
Ll-miRn2	TCACTCCAACTTTGACCTTCT	21	TRINITY_DN50576_c0_g2_i1	215	−84.70	**65-kDa microtubule-associated protein 7**
Ll-miRn3	TGAAGAGGGAGGGAGACTGATG	22	TRINITY_DN77107_c0_g1	185	−86.50	**SRC2 homolog**
Ll-miRn4	GTAGCACCATCAAGATTCACA	21	TRINITY_DN43941_c0_g1_i1	151	−60.30	RCC1 and BTB domain-containing protein 2
Ll-miRn5	TGGAATAGTGAATGAGACATC	21	TRINITY_DN52736_c3_g2_i2	102	−38.70	Probable cinnamyl alcohol dehydrogenase 9
Ll-miRn6	TGCTATCCATCCTGAGTTTCA	21	TRINITY_DN54182_c6_g1_i1	133	−47.90	Probable amino acid permease 7
Ll-miRn7	AGAGGTGTATGGCACAAGAGA	21	TRINITY_DN53175_c1_g6_i1	85	−36.60	Probable protein phosphatase 2C
Ll-miRn8	TGAAGTGTTTGGGGGAACTCC	21	TRINITY_DN44441_c0_g1_i1	102	−37.40	**ATP sulfurylase 1**
Ll-miRn9	TCGGACCAGGCTTTATTCCTT	21	TRINITY_DN50586_c0_g1_i3	167	−65.60	**Homeobox-leucine zipper protein REVOLUTA**
Ll-miRn10	ATGTTGTGATGGGAATCAATG	21	TRINITY_DN67022_c0_g1_i1	84	−43.50	**CBL-interacting serine/threonine-protein kinase 6**
Ll-miRn11	TAAAGACCTCATTCTCTCATG	21	TRINITY_DN31556_c0_g1_i1	130	−62.80	**Vacuolar protein sorting-associated protein 62**
Ll-miRn12	AGGTCATCTTGCAGCTTCAAT	21	TRINITY_DN52990_c2_g1_i5	71	−36.84	DNA-directed RNA polymerase I subunit 1
Ll-miRn15	TTCGGCTTTCTACTTCTCATG	21	TRINITY_DN54101_c8_g2_i10	156	−66.20	**Transcription termination factor MTERF8**
Ll-miRn16	AGTTCTTTAGATGGGCTGGACGCC	24	TRINITY_DN52523_c6_g2_i1	83	−36.50	Amino acid transporter AVT6A
Ll-miRn17	TGTCTCATTCTCTATCTCAAG	21	TRINITY_DN51068_c0_g1_i2	142	−64.30	IST1-like protein
Ll-miRn18	AATAGGGCACATCTCTCATGG	22	TRINITY_DN46596_c0_g1_i1	112	−49.00	E3 ubiquitin-protein ligase HOS1
Ll-miRn19	TCCAAAGGGATCGCATTGATTT	22	TRINITY_DN53637_c4_g2_i4	110	−48.10	**AUXIN SIGNALING F-BOX 3**
Ll-miRn21	TGAGCATGAGGATAAGGACGG	21	TRINITY_DN50271_c0_g3_i1	246	−144.90	**Tetratricopeptide repeat protein 1**
Ll-miRn22	TATCATTCCATACATCGTCTCG	21	TRINITY_DN50592_c0_g4_i2	80	−33.60	Putative disease resistance RPP13-like protein 1
Ll-miRn24	ATTGTCACTGTATCATTCACCATT	24	TRINITY_DN52987_c0_g1_i1	104	−32.30	Zinc finger CCCH domain-containing protein 55
Ll-miRn25	TGGTACAAAAAGTGGGGCAAC	21	TRINITY_DN48871_c3_g1_i9	151	−43.90	Nuclear transcription factor Y subunit A-9
Ll-miRn26	TGTTGTTTTCTGGTAAAAATA	21	TRINITY_DN58488_c1_g2_i4	99	−33.80	Auxin-responsive protein IAA27
Ll-miRn27	ATTAGATCATGTGGCAGTTTCACC	24	TRINITY_DN51506_c3_g2_i5	77	−36.60	U-box domain-containing protein 33
Ll-miRn28	TACGGGTGTCCTCACCTCTGAT	22	TRINITY_DN70730_c0_g1_i1	98	−36.90	**ISWI chromatin-remodeling complex ATPase**
Ll-miRn29	TGGGATAGAGAGTGAGATACC	21	TRINITY_DN51068_c0_g1_i2	125	−67.80	Ethylene-responsive transcription factor ERF017
Ll-miRn30	TTCGTTTGTGTGCAGACTCTGT	22	TRINITY_DN57730_c1_g9_i2	105	−42.70	**Endoribonuclease Dicer homolog 2**
Ll-miRn31	GCGTACCAGGAGCCATGCATG	21	TRINITY_DN58934_c0_g4_i1	149	−60.20	Calcium-transporting ATPase 4
Ll-miRn32	AAGGGTTGTTTACAGAGTTTA	21	TRINITY_DN51330_c0_g1_i1	128	−55.40	26S proteasome regulatory subunit 7

**Table 4:** Expressed miRNAs identified in comparisons of flower development stages between lower and upper parts of the raceme with padj < 0.05.

Flower Development						
ID	miRNA Sequence	miRBase Annotation	log_2_FC	*p*-value	padj	Target Description (psRNAtarget/degradome)
**Lower flowers development**						
LF2 vs LF1						
Ll-miRn22	TATCATTCCATACATCGTCTCG	new	0.71	0.0000	0.0004	Putative disease resistance RPP13-like protein 1
Ll-miR401	TTGACAGAAGAGAGTGAGCAC	gma-miR156k	0.61	0.0003	0.0032	**Squamosa promoter-binding-like protein 2**
Ll-miR265	TCGGACCAGGCTTCATTCCTT	ata-miR166c-3p	0.61	0.0001	0.0007	**Homeobox-leucine zipper protein ATHB-15**
Ll-miRn27	ATTAGATCATGTGGCAGTTTCACC	new	0.51	0.0028	0.0241	U-box domain-containing protein 33
Ll-miR454	TTTGGATTGAAGGGAGCTCTT	aly-miR159b-3p	0.47	0.0000	0.0000	Transcription factor GAMYB
Ll-miR124	TGACAGAAGAGAGTGAGCAC	ama-miR156	0.43	0.0021	0.0193	**Squamosa promoter-binding-like protein 13B**
Ll-miR247	TCGCTTGGTGCAGGTCGGGAA	aly-miR168a-5p	−0.68	0.0000	0.0000	AGO1
Ll-miR102	CGCTGTCCATCCTGAGTTTCA	bra-miR390-3p	−0.69	0.0000	0.0003	TAS3
Ll-miR115	CTCGTTGTCTGTTCGACCTTG	ppe-miR858	−0.76	0.0000	0.0001	**Transcription repressor MYB5**
Ll-miR53	ATCATGCTATCCCTTTGGATT	gma-miR393c-3p	−1.02	0.0034	0.0278	**Midasin-like**
Ll-miR82	CCCGCCTTGCATCAACTGAAT	aly-miR168a-3p	−1.31	0.0000	0.0000	FAD-linked sulfhydryl oxidase ERV1
Ll-miRn10	ATGTTGTGATGGGAATCAATG	new	−1.37	0.0000	0.0000	**CBL-interacting Ser/Thr -protein kinase 6**
Ll-miRn3	TGAAGAGGGAGGGAGACTGATG	new	−1.43	0.0006	0.0060	**SRC2 homolog**
Ll-miR198	GTTCAATAAAGCTGTGGGAA	osa-miR396a-3p	−1.75	0.0000	0.0000	ECERIFERUM 1
Ll-miRn31	GCGTACCAGGAGCCATGCATG	new	−1.99	0.0001	0.0007	Calcium-transporting ATPase 4
Ll-miR200	GTTCAATAAAGCTGTGGGAAG	aly-miR396a-3p	−2.00	0.0000	0.0000	ECERIFERUM 1
Ll-miR155	GCTCAAGAAAGCTGTGGGAGA	gma-miR396b-3p	−2.04	0.0000	0.0000	**Lysine-specific demethylase JMJ25**
Ll-miR199	GTTCAATAAAGCTGTGGGAAA	ata-miR396e-3p	−2.31	0.0000	0.0000	ECERIFERUM 1
LF3 vs LF2						
Ll-miR258	TCGGACCAGGCTTCATTCCCG	cpa-miR166d	0.91	0.0000	0.0001	Homeobox-leucine zipper protein ATHB-15
Ll-miRn1	TTGCCAATTCCACCCATGCCTA	new	0.87	0.0000	0.0008	**SUPPRESSOR OF GENE SILENCING 3**
Ll-miR9	AAGCTCAGGAGGGATAGCGCC	aly-miR390a-5p	0.78	0.0005	0.0100	**TAS3**
Ll-miR118	CTGAAGTGTTTGGGGGAACTC	aly-miR395d-3p	0.70	0.0000	0.0007	**ATP sulfurylase 1, chloroplastic**
Ll-miR264	TCGGACCAGGCTTCATTCCTC	aqc-miR166a	0.66	0.0037	0.0401	**Homeobox-leucine zipper protein ATHB-15**
Ll-miRn22	TATCATTCCATACATCGTCTCG	new	0.46	0.0032	0.0401	Putative disease resistance RPP13-like protein 1
Ll-miR384	TTCCACAGCTTTCTTGAACTT	aly-miR396b-5p	−0.35	0.0033	0.0401	MPE-cyclase
Ll-miR115	CTCGTTGTCTGTTCGACCTTG	ppe-miR858	−0.59	0.0048	0.0426	**Transcription repressor MYB5**
Ll-miR200	GTTCAATAAAGCTGTGGGAAG	aly-miR396a-3p	−0.62	0.0000	0.0000	ECERIFERUM 1
Ll-miR108	CGTGTTCTCAGGTCGCCCCTG	ppe-miR398b	−1.08	0.0044	0.0426	Plastocyanin
Ll-miR60	ATGCACTGCCTCTTCCCTGGC	ahy-miR408-3p	−1.09	0.0024	0.0385	**Basic blue protein**
LF4 vs LF3						
ND						
**Upper flowers development**						
UF2 vs UF1						
Ll-miR119	TGAAGTGTTTGGGGGAACTCC	sly-miR395a	1.22	0.0000	0.0019	**ATP sulfurylase 1, chloroplastic**
Ll-miR452	TTTGGATTGAAGGGAGCTCTC	lus-miR159b	0.74	0.0001	0.0019	**Gamma-glutamyl peptidase 5**
Ll-miR118	CTGAAGTGTTTGGGGGAACTC	aly-miR395d-3p	0.64	0.0001	0.0019	**ATP sulfurylase 1, chloroplastic**
Ll-miR177	GGAATGTTGTCTGGCTCGAGG	aly-miR166a-5p	0.50	0.0002	0.0033	Transcription factor RADIALIS
Ll-miR174	GGAATGTTGGCTGGCTCGAGG	mtr-miR166e-5p	−0.45	0.0002	0.0033	**Nucleolar GTP-binding protein 1**
Ll-miR285	TGAAGCTGCCAGCATGATCTTA	mdm-miR167h	−0.56	0.0023	0.0286	**Auxin response factor 6**
Ll-miRn10	ATGTTGTGATGGGAATCAATG	new	−0.96	0.0008	0.0117	**CBL-interacting Ser/Thr-protein kinase 6**
Ll-miR155	GCTCAAGAAAGCTGTGGGAGA	gma-miR396b-3p	−1.17	0.0000	0.0000	**Lysine-specific demethylase JMJ25**
UF3 vs UF2						
Ll-miR258	TCGGACCAGGCTTCATTCCCG	cpa-miR166d	0.70	0.0022	0.0323	Homeobox-leucine zipper protein ATHB-15
Ll-miR247	TCGCTTGGTGCAGGTCGGGAA	aly-miR168a-5p	−0.40	0.0005	0.0113	AGO1
Ll-miR102	CGCTGTCCATCCTGAGTTTCA	bra-miR390-3p	−0.96	0.0000	0.0004	TAS3
Ll-miR199	GTTCAATAAAGCTGTGGGAAA	ata-miR396e-3p	−0.97	0.0000	0.0001	ECERIFERUM 1
Ll-miR60	ATGCACTGCCTCTTCCCTGGC	ahy-miR408-3p	−1.01	0.0008	0.0145	**Basic blue protein**
Ll-miR200	GTTCAATAAAGCTGTGGGAAG	aly-miR396a-3p	−1.02	0.0000	0.0000	ECERIFERUM 1
Ll-miR100	CGCTATCCATCCTGAGTTTCA	aly-miR390a-3p	−1.10	0.0001	0.0021	TAS3
Ll-miR99	CGCTATCCATCCTGAGTTTC	gma-miR390a-3p	−1.16	0.0014	0.0228	TAS3
Ll-miR53	ATCATGCTATCCCTTTGGATT	gma-miR393c-3p	−1.19	0.0001	0.0017	**Midasin-like**
Ll-miR155	GCTCAAGAAAGCTGTGGGAGA	gma-miR396b-3p	−1.45	0.0000	0.0000	**Lysine-specific demethylase JMJ25**
UF4 vs UF3						
Ll-miR438	TTGTGTTCTCAGGTCACCCCT	stu-miR398b-3p	1.09	0.0003	0.0098	**Probable nucleoredoxin 1**
Ll-miR281	TGAAGCTGCCAGCATGATCTGA	ata-miR167b-5p	0.92	0.0001	0.0048	Auxin response factor 6 and ARF8
Ll-miR445	TTTGGACTGAAGGGAGCTCCT	atr-miR319b	0.84	0.0000	0.0009	Transcription factor TCP4
Ll-miR9	AAGCTCAGGAGGGATAGCGCC	aly-miR390a-5p	0.78	0.0004	0.0098	**TAS3**
Ll-miR346	TGGAGAAGCAGGGCACGTGCA	aly-miR164a-5p	0.73	0.0029	0.0359	CUP-SHAPED COTYLEDON 2
Ll-miR280	TGAAGCTGCCAGCATGATCTG	atr-miR167	0.66	0.0010	0.0207	Auxin response factor 6 and ARF8
Ll-miR130	CTTGGACTGAAGGGAGCTCCC	ppt-miR319c	0.65	0.0001	0.0034	**Transcription factor MYB33**
Ll-miR115	CTCGTTGTCTGTTCGACCTTG	ppe-miR858	−0.67	0.0019	0.0333	**Transcription repressor MYB5**
Ll-miR100	CGCTATCCATCCTGAGTTTCA	aly-miR390a-3p	−0.70	0.0025	0.0337	TAS3
Ll-miRn11	TAAAGACCTCATTCTCTCATG	new	−0.83	0.0037	0.0386	**Vacuolar protein sorting-associated protein 62**
Ll-miR99	CGCTATCCATCCTGAGTTTC	gma-miR390a-3p	−0.86	0.0038	0.0386	TAS3
Ll-miR102	CGCTGTCCATCCTGAGTTTCA	bra-miR390-3p	−0.91	0.0025	0.0337	TAS3

**Table 5:** Differentially expressed miRNAs identified in comparisons between flowers from lower and upper parts of the raceme with padj < 0.05.

Flowers From Upper and Lower Parts of Receme						
ID	miRNA sequence	miRBase annotation	log_2_FC	*p*-value	padj	Target description (psRNAtarget/degradome)
UF1 vs LF1						
Ll-miR281	TGAAGCTGCCAGCATGATCTGA	ata-miR167b-5p	−0.47	0.0002	0.0054	Auxin response factor 6 and ARF8
Ll-miRn30	TTCGTTTGTGTGCAGACTCTGT	new	−0.49	0.0009	0.0221	**Endoribonuclease Dicer homolog 2**
Ll-miR118	CTGAAGTGTTTGGGGGAACTC	aly-miR395d-3p	−0.58	0.0000	0.0000	**ATP sulfurylase 1, chloroplastic**
Ll-miR102	CGCTGTCCATCCTGAGTTTCA	bra-miR390-3p	−0.71	0.0000	0.0000	TAS3
Ll-miRn29	TGGGATAGAGAGTGAGATACC	new	−1.00	0.0000	0.0000	Ethylene-responsive transcription factor ERF017
Ll-miR94	CGATGTTGGTGAGGTTCAATC	gma-miR171k-5p	−1.09	0.0012	0.0253	Transcription factor MYB4
Ll-miR198	GTTCAATAAAGCTGTGGGAA	osa-miR396a-3p	−1.10	0.0000	0.0001	ECERIFERUM 1
Ll-miR82	CCCGCCTTGCATCAACTGAAT	aly-miR168a-3p	−1.25	0.0000	0.0000	FAD-linked sulfhydryl oxidase ERV1
Ll-miR200	GTTCAATAAAGCTGTGGGAAG	aly-miR396a-3p	−1.44	0.0000	0.0000	ECERIFERUM 1
Ll-miR199	GTTCAATAAAGCTGTGGGAAA	ata-miR396e-3p	−1.47	0.0000	0.0000	ECERIFERUM 1
Ll-miRn25	TGGTACAAAAAGTGGGGCAAC	new	−1.82	0.0001	0.0030	Nuclear transcription factor Y subunit A-9
Ll-miRn3	TGAAGAGGGAGGGAGACTGATG	new	−2.11	0.0000	0.0000	**SRC2 homolog**
UF2 vs LF2						
Ll-miR433	TTGGCATTCTGTCCACCTCC	ahy-miR394	3.11	0.0000	0.0000	**F-box only protein 6**
Ll-miR341	TGGACTGAAGGGAGCTCCTTC	gma-miR319q	3.07	0.0000	0.0000	**Transcription factor TCP2**
Ll-miR424	TTGGACTGAAGGGAGCTCCCT	aau-miR319	1.98	0.0000	0.0000	Transcription factor TCP4
Ll-miR423	TTGGACTGAAGGGAGCTCCCA	mtr-miR319c-3p	1.91	0.0000	0.0000	Transcription factor TCP4
Ll-miR224	TCCAAAGGGATCGCATTGATCC	aly-miR393a-5p	1.88	0.0002	0.0052	**TRANSPORT INHIBITOR RESPONSE 1**
Ll-miR131	TTGGACTGAAGGGAGCTCCT	mes-miR319h	1.78	0.0000	0.0000	Transcription factor TCP2
Ll-miR332	TGCCTGGCTCCCTGTATGCC	bdi-miR160f	1.63	0.0015	0.0262	**Auxin response factor 18**
Ll-miR130	TTGGACTGAAGGGAGCTCCC	ppt-miR319c	1.60	0.0000	0.0000	**Transcription factor MYB33**
Ll-miR329	TGCCTGGCTCCCTGAATGCCA	ahy-miR160-5p	1.56	0.0024	0.0381	**Auxin response factor 16**
Ll-miR199	GTTCAATAAAGCTGTGGGAAA	ata-miR396e-3p	0.66	0.0005	0.0095	ECERIFERUM 1
Ll-miR200	GTTCAATAAAGCTGTGGGAAG	aly-miR396a-3p	0.31	0.0001	0.0030	ECERIFERUM 1
UF3 vs LF3						
Ll-miR433	TTGGCATTCTGTCCACCTCC	ahy-miR394	2.59	0.0000	0.0009	**F-box only protein 6**
Ll-miR333	TGCCTGGCTCCCTGTATGCCA	aly-miR160a-5p	2.18	0.0002	0.0072	**Auxin response factor 18**
Ll-miR332	TGCCTGGCTCCCTGTATGCC	bdi-miR160f	1.88	0.0006	0.0210	**Auxin response factor 18**
Ll-miR224	TCCAAAGGGATCGCATTGATCC	aly-miR393a-5p	1.87	0.0018	0.0498	**TRANSPORT INHIBITORRESPONSE 1**
Ll-miR115	CTCGTTGTCTGTTCGACCTTG	ppe-miR858	0.78	0.0001	0.0072	**Transcription repressor MYB5**
Ll-miR453	TTTGGATTGAAGGGAGCTCTG	bdi-miR159b-3p	−0.57	0.0002	0.0072	**RING-type zinc-finger**
Ll-miR229	TCCACAGGCTTTCTTGAACTG	ata-miR396a-5p	−1.94	0.0013	0.0404	**Growth-regulating factor 5**
Ll-miR432	TTGGATTTTTATTTAGGACGG	osa-miR5490	−2.29	0.0001	0.0072	**Acid phosphatase 1**
Ll-miR311	TGAGGAATCACTAGTAGTCGT	osa-miR5794	−2.32	0.0001	0.0072	**Uncharacterized WD repeat-containing protein**
Ll-miR92	CGATCTTGAGGCAGGAACTGAG	osa-miR1861b	−4.11	0.0000	0.0000	Clathrin interactor EPSIN 2
Ll-miR251	TCGGACCAGGCTTCAATCCCT	ata-miR5168-3p	−5.13	0.0000	0.0000	Homeobox-leucine zipper protein ATHB-15
UF4 vs LF4						
Ll-miR445	TTTGGACTGAAGGGAGCTCCT	atr-miR319b	0.75	0.0001	0.0078	Transcription factor TCP4
Ll-miR100	CGCTATCCATCCTGAGTTTCA	aly-miR390a-3p	−0.97	0.0006	0.0452	TAS3

**Table 6:** Differentially expressed miRNAs identified in comparisons between pedicels collected from abscised or non-abscised flowers (FPAB vs FPNAB) with padj < 0.05.

Flower Pedicels						
ID	miRNA sequence	miRBase annotation	log_2_FC	*p*-value	padj	Target description (psRNAtarget/degradome)
FPAB vs FPNAB						
Ll-miR281	TGAAGCTGCCAGCATGATCTGA	ata-miR167b-5p	4.77	0.0000	0.0000	Auxin response factor 6 and ARF8
Ll-miR39	AGATCATGTGGCAGTTTCACC	ahy-miR167-3p	4.65	0.0000	0.0000	Transcription repressor OFP14
Ll-miR280	TGAAGCTGCCAGCATGATCTG	atr-miR167	3.73	0.0000	0.0000	Auxin response factor 6 and ARF8
Ll-miR283	TGAAGCTGCCAGCATGATCTGG	aly-miR167d-5p	3.19	0.0000	0.0000	Auxin response factor 6 and ARF8
Ll-miR276	TGAAGCTGCCAGCATGATCT	bna-miR167d	2.74	0.0000	0.0005	Auxin response factor 6 and ARF8
Ll-miRn18	AATAGGGCACATCTCTCATGG	new	2.26	0.0001	0.0006	E3 ubiquitin-protein ligase HOS1
Ll-miR193	GTCGTTGTAGTATAGTGG	gma-miR6300	2.18	0.0001	0.0006	-
Ll-miR438	TTGTGTTCTCAGGTCACCCCT	stu-miR398b-3p	2.09	0.0000	0.0000	**Probable nucleoredoxin 1**
Ll-miR325	TGCCAAAGGAGAGTTGCCCTG	aly-miR399b-3p	1.95	0.0000	0.0000	Inorganic phosphate transporter 1–4
Ll-miR364	TGTGTTCTCAGGTCACCCCTT	aly-miR398a-3p	1.94	0.0010	0.0071	**Superoxide dismutase [Cu-Zn]**
Ll-miR415	TTGAGCCGTGCCAATATCACG	aly-miR171b-3p	1.80	0.0017	0.0117	**Scarecrow-like protein 6**
Ll-miR366	TGTGTTCTCAGGTCGCCCCTG	aqc-miR398b	1.66	0.0005	0.0042	Superoxide dismutase [Cu-Zn]
Ll-miR260	TCGGACCAGGCTTCATTCCCT	csi-miR166d	1.07	0.0078	0.0450	**Homeobox-leucine zipper protein ATHB-14**
Ll-miR273	TCTCGTTGTCTGTTCGACCTT	cme-miR858	1.02	0.0037	0.0223	**Transcription factor MYB78**
Ll-miR402	TTGACAGAAGATAGAGAGC	hbr-miR156	−0.89	0.0008	0.0060	Squamosa promoter-binding protein 1
Ll-miRn21	TGAGCATGAGGATAAGGACGG	new	−1.18	0.0000	0.0000	**Tetratricopeptide repeat protein 1**
Ll-miR198	GTTCAATAAAGCTGTGGGAA	osa-miR396a-3p	−1.24	0.0024	0.0159	ECERIFERUM 1
Ll-miRn5	TGGAATAGTGAATGAGACATC	new	−1.25	0.0003	0.0024	Probable cinnamyl alcohol dehydrogenase 9
Ll-miR102	CGCTGTCCATCCTGAGTTTCA	bra-miR390-3p	−1.27	0.0003	0.0027	TAS3
Ll-miR199	GTTCAATAAAGCTGTGGGAAA	ata-miR396e-3p	−1.49	0.0005	0.0039	ECERIFERUM 1
Ll-miR200	GTTCAATAAAGCTGTGGGAAG	aly-miR396a-3p	−1.61	0.0001	0.0009	ECERIFERUM 1
Ll-miR44	AGCTGCTGACTCGTTGGTTCA	mtr-miR4414a-5p	−1.76	0.0012	0.0084	Non-specific phospholipase C1
Ll-miR151	GCGTATGAGGAGCCAAGCATA	gma-miR160a-3p	−1.90	0.0007	0.0055	E3 ubiquitin-protein ligase RFWD3
Ll-miR57	TGAAGTGTTTGGGGGAACTC	gma-miR395i	−2.01	0.0000	0.0000	**ATP sulfurylase 1, chloroplastic**
Ll-miR170	GGAACGTTGGCTGGCTCGAGG	ata-miR166c-5p	−2.02	0.0072	0.0425	Probable methyltransferase PMT21
Ll-miR119	TGAAGTGTTTGGGGGAACTCC	sly-miR395a	−2.23	0.0000	0.0000	**ATP sulfurylase 1, chloroplastic**
Ll-miRn3	TGAAGAGGGAGGGAGACTGATG	new	−2.26	0.0027	0.0171	**SRC2 homolog**
Ll-miR118	CTGAAGTGTTTGGGGGAACTC	aly-miR395d-3p	−2.47	0.0000	0.0000	**ATP sulfurylase 1, chloroplastic**
Ll-miR99	CGCTATCCATCCTGAGTTTC	gma-miR390a-3p	−2.65	0.0001	0.0006	TAS3
Ll-miR100	CGCTATCCATCCTGAGTTTCA	aly-miR390a-3p	−2.75	0.0000	0.0000	TAS3
Ll-miR162	GCTCTCTAAGCTTCTGTCATCA	aly-miR157b-3p	−2.90	0.0000	0.0003	Dr1 homolog
Ll-miR94	CGATGTTGGTGAGGTTCAATC	gma-miR171k-5p	−3.01	0.0000	0.0000	Transcription factor MYB4
Ll-miRn32	AAGGGTTGTTTACAGAGTTTA	new	−3.18	0.0000	0.0000	26S proteasome regulatory subunit 7
Ll-miR174	GGAATGTTGGCTGGCTCGAGG	mtr-miR166e-5p	−3.46	0.0000	0.0000	**Nucleolar GTP-binding protein 1**

## Supplementary Materials

Supplementary materials can be found at https://www.mdpi.com/1422-0067/20/20/5122/s1.

## Abbreviations

AZ	abscission zone
DE	differentially expressed/differential expression
FPAB	flower pedicel abscissed
FPKM	fragments per kilobase of transcript per million mapped reads
FPNAB	flower pedicel non abscissed
GO	gene ontology
KEGG	kyoto encyclopedia of genes and genomes
LF	lower flower
miRNAs	microRNAs
ncRNA	non coding RNA
NGS	next generation sequencing
Pajd	adjusted p-value
RPM	reads per million
siRNA	small interfering RNA
sRNA	small RNA
UF	upper flower
UFR	upper flower after removing lower flowers
