# Supplementary material for: Integrated Analysis of Small RNA, Transcriptome and Degradome Sequencing Provides New Insights into Floral Development and Abscission in Yellow Lupine (Lupinus luteus L.)
Source: Int J Mol Sci. 2019 Oct 16;20(20):5122. doi: 10.3390/ijms20205122 (PMC6854478; doi:10.3390/ijms20205122)
Supplement: Supplementary file 1 [file ijms-20-05122-s001.zip › ijms-594387-SI/ijms-594387-SI.pdf]

International Journal of
Molecular Sciences

MDPI

Supplementary Materials:

Additional file 1: Table S1. Sample description and the numbers of the identified miRNAs in the particular sRNA libraries. **Table S2**. List of known miRNAs identified in all small RNA libraries. **Table S3.** *De novo* transcriptome assembly statistics. **Table S4.** List of novel miRNAs identified in *Lupinus luteus* and their expression. **Table S5.** Expression of the identified miRNAs in RPM (reads per million). **Table S6.** List of phased siRNAs in *Lupinus luteus* identified with small RNA-seq**. Table S7.** Expression of identified phased siRNAs in RPM (reads per million). **Table S8.** Differentially expressed phased siRNA from all comparisons with padj < 0.05, and description of their targets identified through degradome and psRNATarget analyses. **Table S9.** Comparison of the presence of miRNAs identified in *Lupinus luteus*.

Additional file 2: Table S10. List of stem-loop reverse transcription (SL-RT) and qRT-PCR primer sequences for miRNA, siRNA and targeted genes expression analysis. **Table S11**. **Statistics of degradome sequencing data**. (**a**) Length and quality of reads. (**b**) Quantity of target genes identified for miRNAs and siRNAs in the particular categories. **Table S12.** List and annotation of targets of known and novel miRNAs identified through degradome sequencing with p-value < 0.5. **Table S13.** List and annotation of targets of known and novel miRNAs identified using psRNATarget. **Table S14.** List and annotation of phased siRNAs targets identified through degradome sequencing with p-value < 0.5. **Table S15.** List and annotation of phased siRNAs targets identified using psRNATarget. **Table S16.** KEGG analysis of targets of known and novel miRNAs. **Table S17.** GO analysis of targets of known and novel miRNAs. **Table S18**. Expression of elements of the miR390/tasiR-ARF/TAS3/ARFs module.

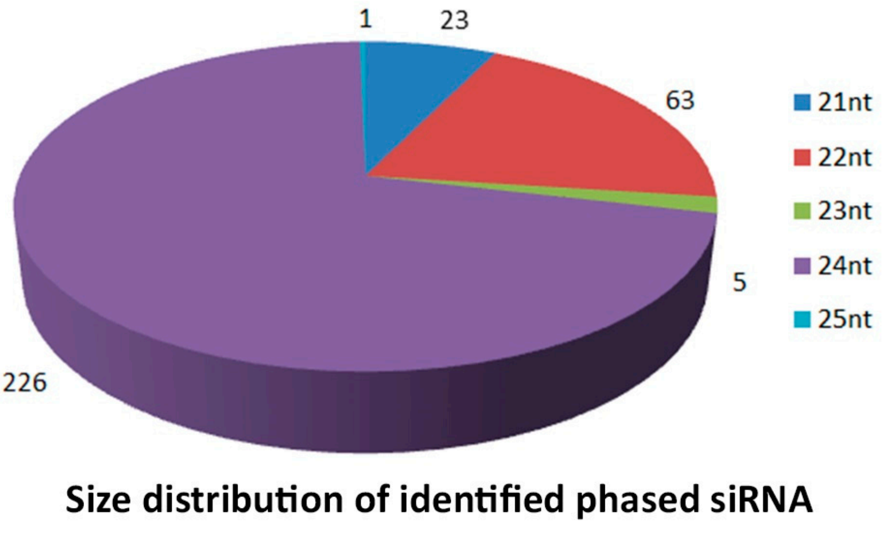

Figure S1. Size distribution of identified phased siRNA.

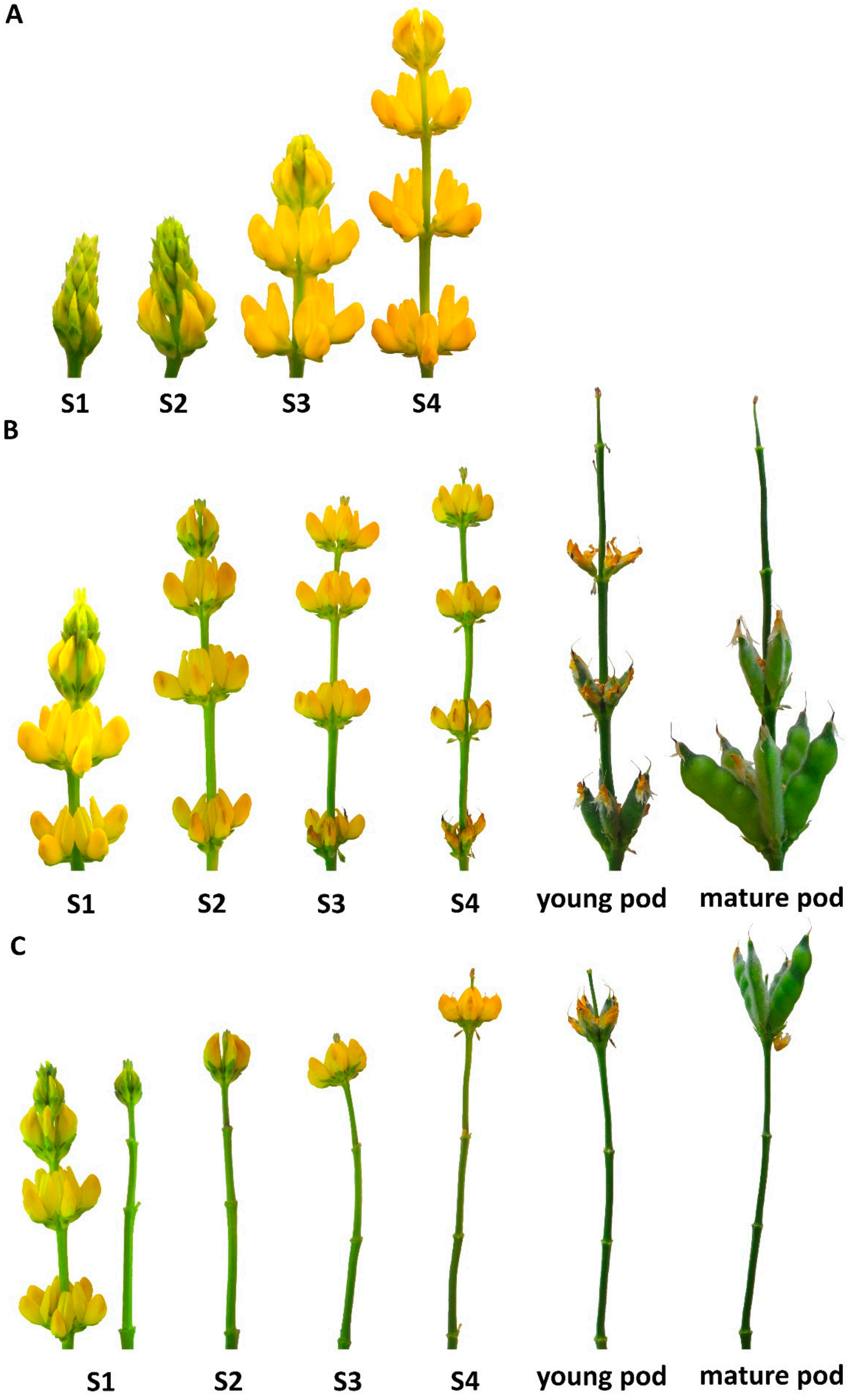

Figure S2. Development of yellow lupine inflorescences from stages S1 to S4. (**A**) In the lower whorls of the inflorescence. (**B**) In the upper whorls of the inflorescence (**C**) In the upper whorls after removing other flowers. Comparison of locations of developing pods in control flowers (B) and after removal of lower flowers (C).

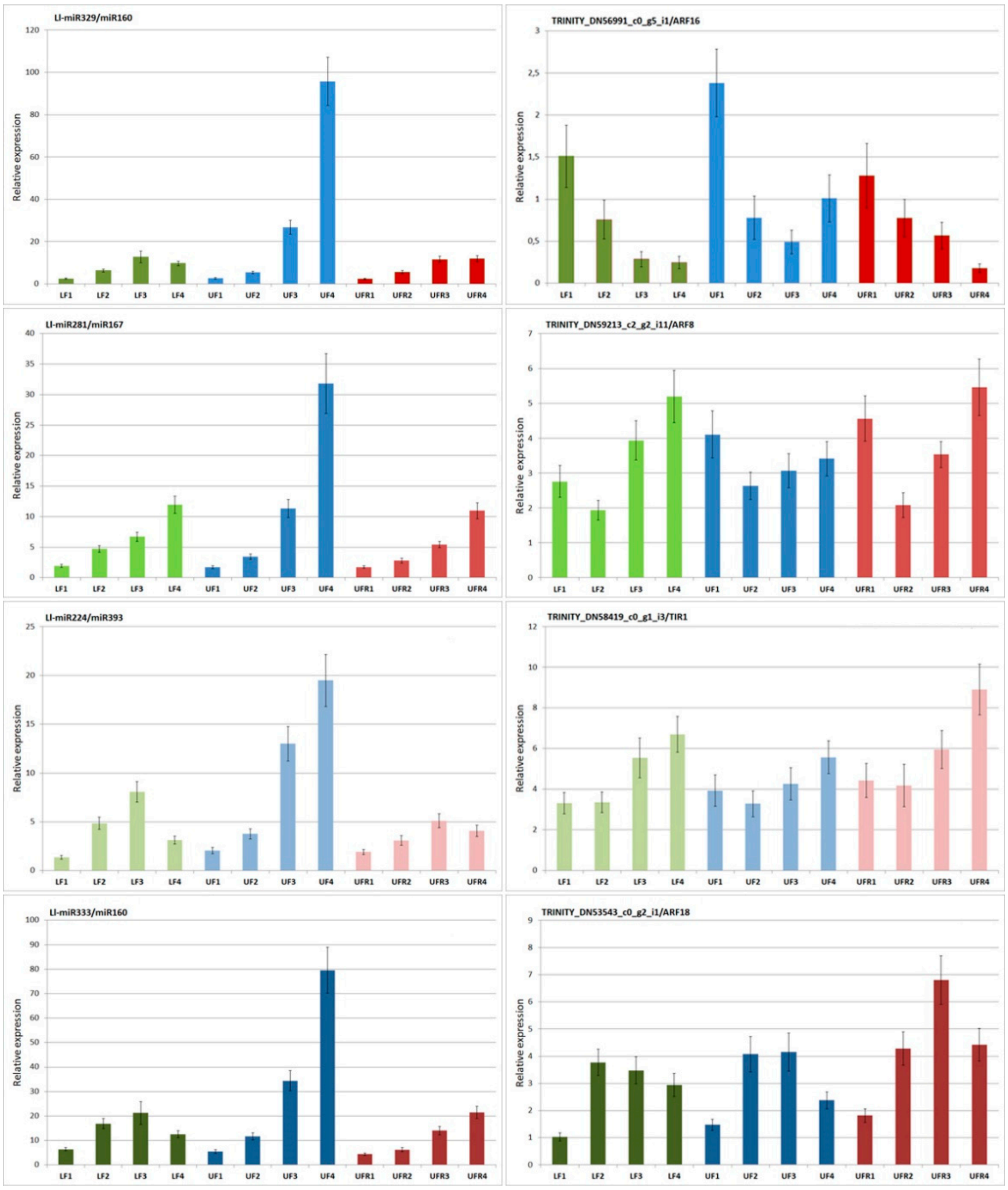

Figure S3. Expression of selected miRNAs and their target genes in yellow lupine during the development of upper (UF), lower (LF) and upper flowers after removal of the lower flowers (UFR). The level of expression examined by RT-qPCR. Vertical bars indicate standard errors.

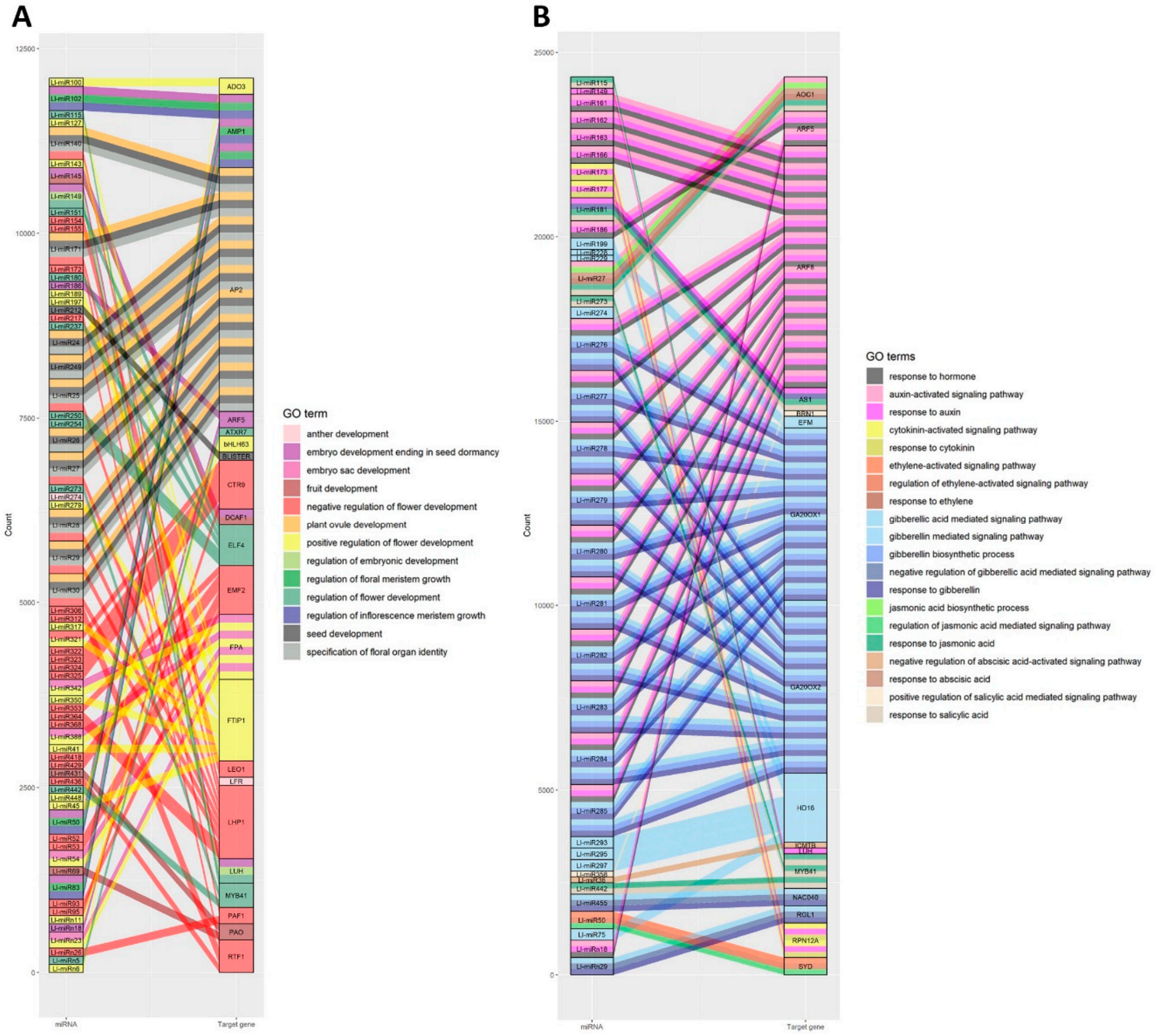

Figure S4. Alluvial diagrams showing participation of *Lupinus luteus* miRNAs in processes categorized to selected GO terms associated with (**a**) plant hormones or (**b**) flower development via regulation of their target genes.

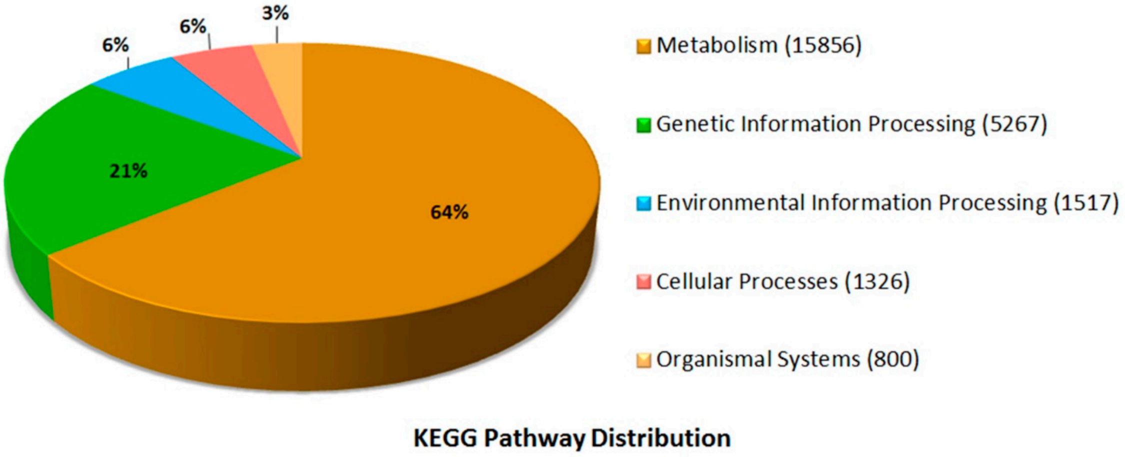

Figure S5. Distribution of miRNA targets across the KEGG biological categories. Numbers of miRNA targets belonging to each category are shown in brackets.

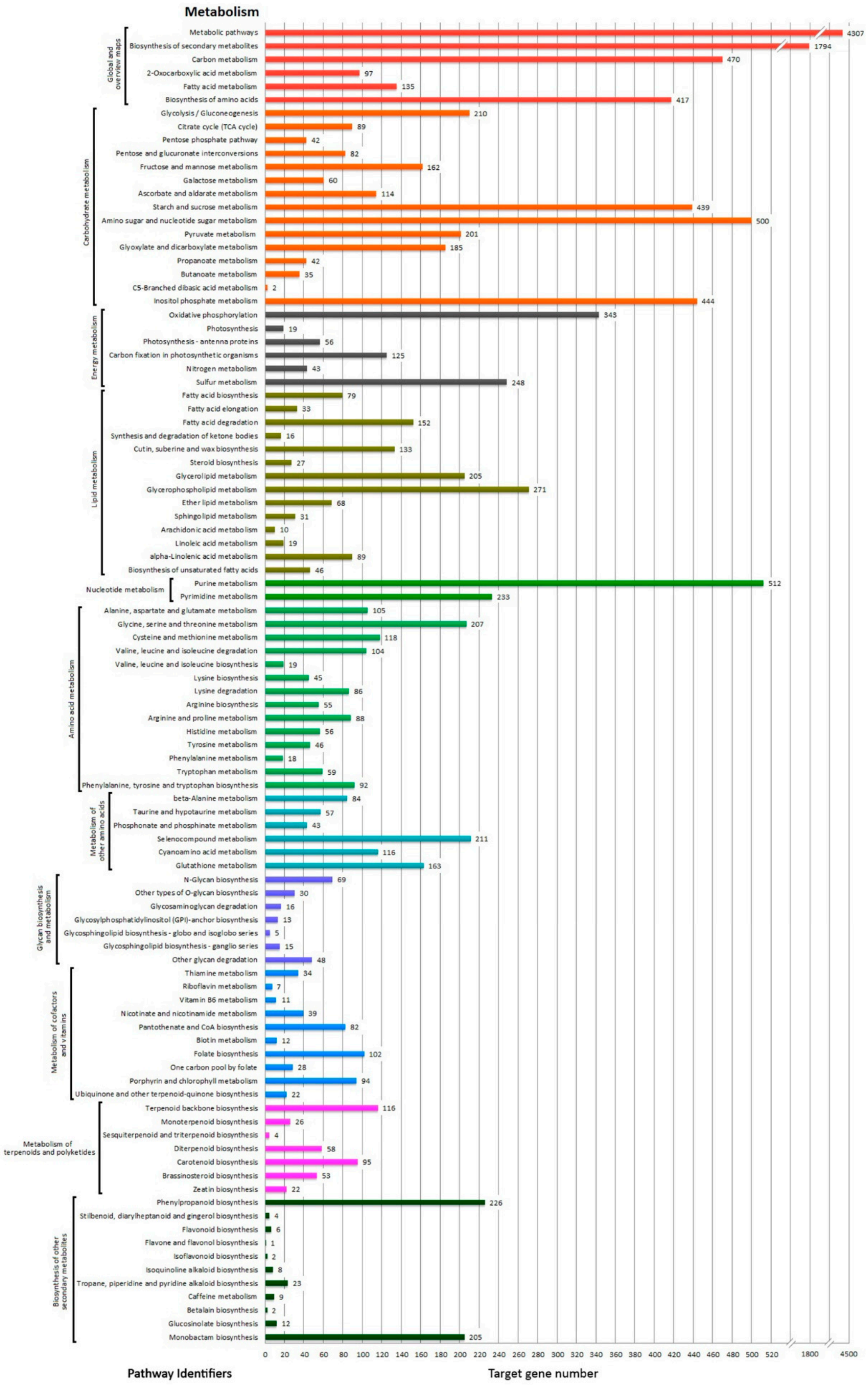

Figure S6. Classification of miRNAs targets into the KEGG "Metabolism" category. Exact number of miRNAs associated with specific category is featured at the end of each column.

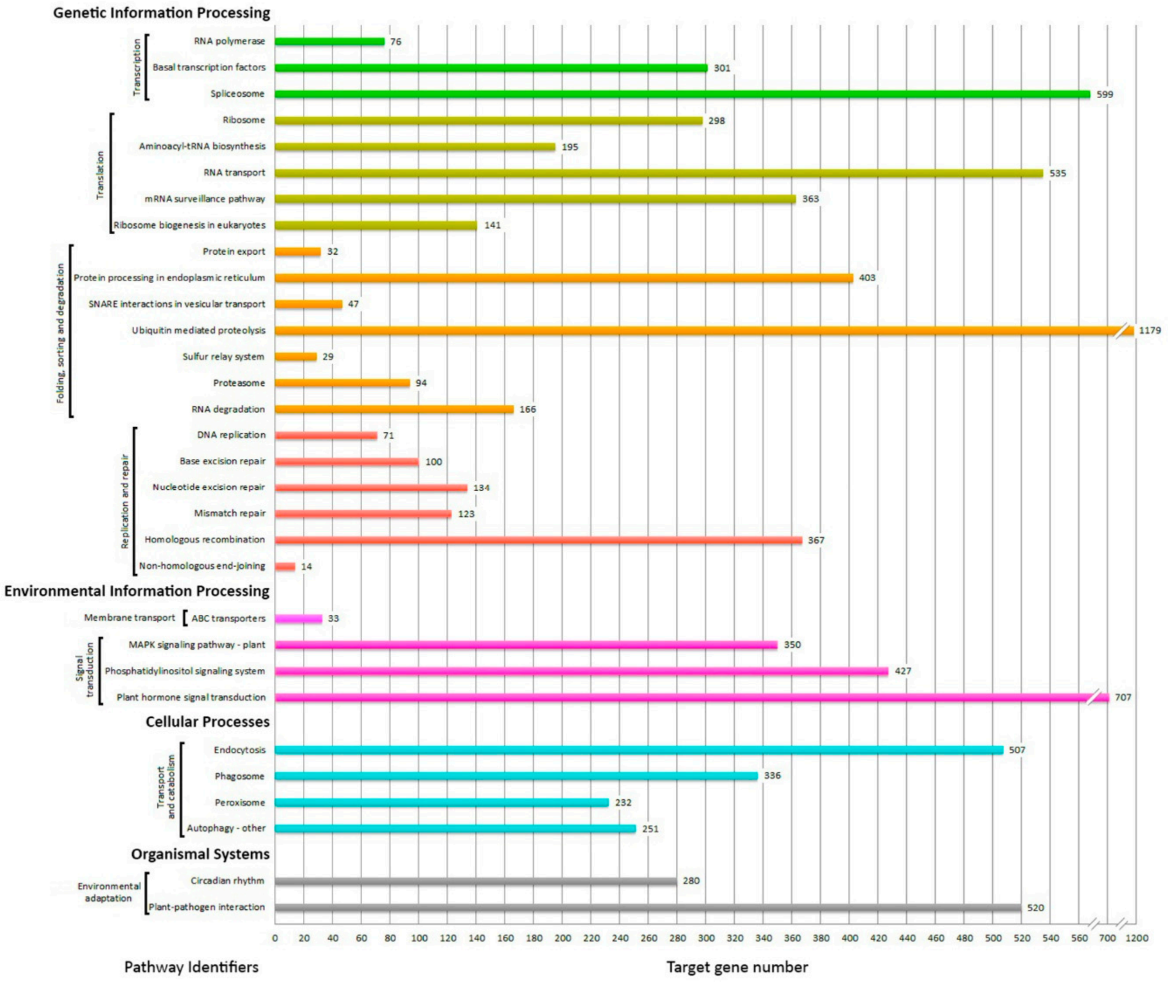

Figure S7. Classification of miRNAs targets into the KEGG "Genetic information processing", "Environmental information processing", "Cellular processes" and "Organismal systems" categories. Exact number of miRNAs associated with specific category is featured at the end of each column.

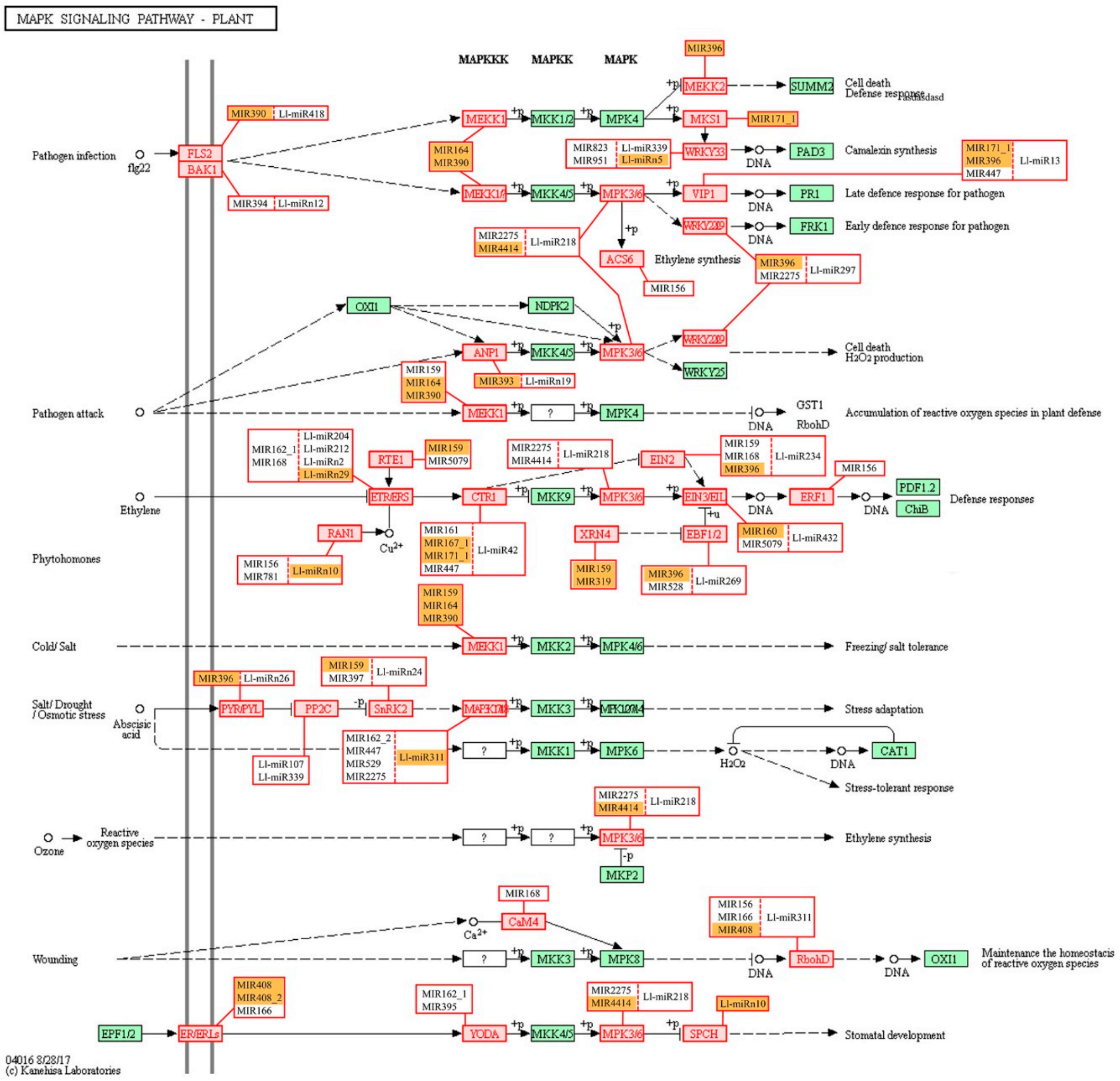

Figure S8. MAPK signal transduction pathway targeted by known and novel miRNAs. Orange indicates DE miRNAs.

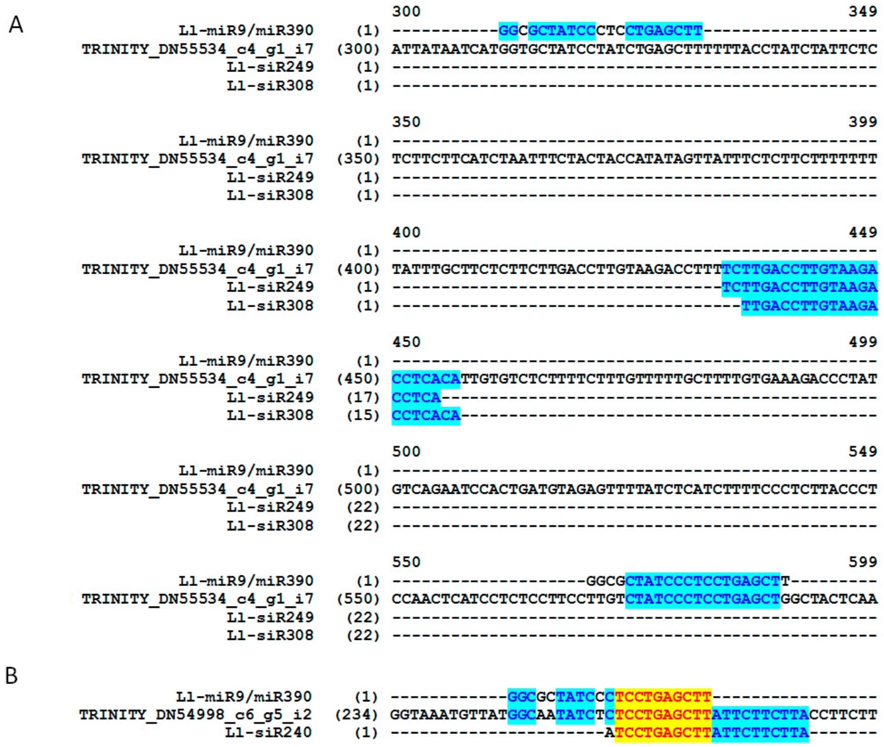

Figure S9. Alignment of TAS gene transcripts with two (**a**) and one (**b**) miR390 target sites.

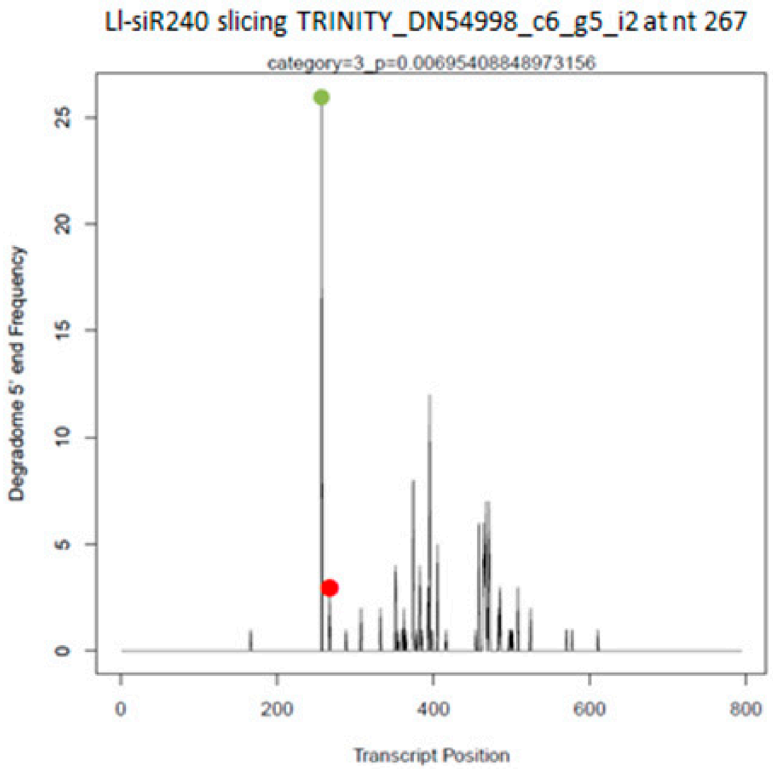

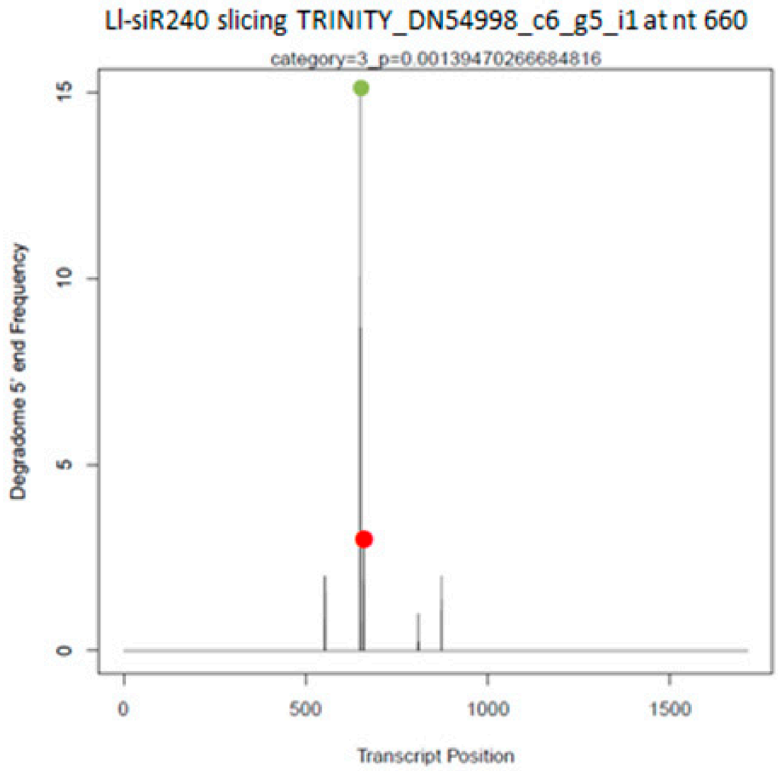

Figure S10. T-plots of the Ll-siR240 target confirmed by degradome sequencing. The T-plots show the distribution of the degradome tags along the full length of the target RNA sequence. The siR240 cleavage site on the transcript is marked with a red dot and the one for miR390 with a green dot.
